# *Sesamum indicum*-derived valdiate as a novel neuroprotective agent targeting PDE10A2 and SIRT1 in Huntington’s disease

**DOI:** 10.3389/fbinf.2026.1868337

**Published:** 2026-08-24

**Authors:** Mukul Shyam, M. D. Wafi Ismail, Deepak Sharma, Prathap Srirangan, Rajiniraja Muniyan, Sabina Evan Prince

**Affiliations:** School of Bio Sciences and Technology, VIT University, Vellore, India

**Keywords:** Huntington’s disease, in-silico, PDE10A2, phytochemical, SIRT 1

## Abstract

**Background:**

Huntington’s disease is prevalent globally, with approximately 4.88 cases per 100,000 people, based on a systematic review and meta-analysis of 33 studies published between 2010 and 2022. Despite its significant prevalence, no proper treatment is available that directly addresses Huntington’s disease. The existing treatments focus on symptom management, such as controlling chorea and psychiatric symptoms. The drugs used for this purpose may also cause side effects, including depression and Parkinson’s disease.

**Methods:**

An integrated experimental and computational approach was employed, involving cold maceration extraction of *Sesamum indicum*, LC-MS/MS based phytochemical profiling, ADMET screening, molecular docking, molecular dynamics simulations, and MMBPSA binding free energy analysis to identify potential inhibitors of Huntington’s diseases associated targets.

**Results:**

Among the LC-MS/MS identified compounds, eight compounds satisfied the ADMET criteria. Valdiate (PubChem CID: 129715809) demonstrated favourable multi-target binding with docking score of −7.3 kcal/mol against HDAC4 and -8.9 kcal/mol against HDAC7. Molecular dynamics simulations confirmed stable protein-ligand interactions, while MMPBSA analysis yielded binding free energies of −23.22 ± 2.45 kcal/mol (HDAC4) and −28.27 ± 2.28 kcal/mol (HDAC7), identifying Valdiate as the most promising potential inhibitor.

**Conclusion:**

Valdiate may serve as a potential inhibitor of mutant huntingtin-associated pathological pathways, pending further *in-vitro* and *in-vivo* validation.

## Graphical Abstract

**Figure:**
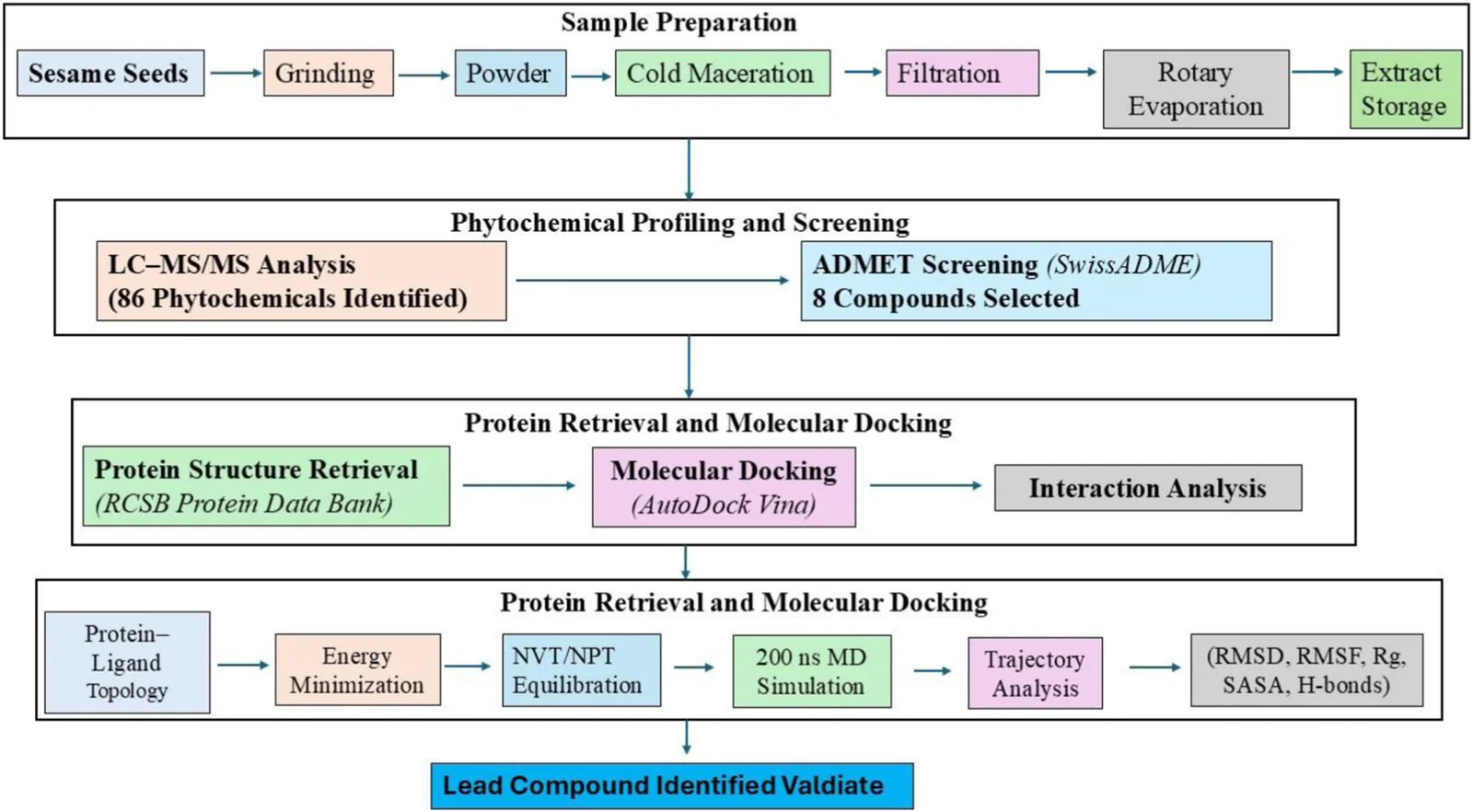

## 1 Background

Huntington’s disease (HD) is a rare genetic neurodegenerative condition that causes motor disability, psychological abnormalities, and cognitive deterioration. Huntington’s disease is caused by the presence of a trinucleotide CAG repetition mutation in the HTT gene located in chromosome 4. The mutation results in the synthesis of an abnormal huntingtin protein that forms aggregates causing neurological damage and cell death mainly in the basal ganglia and cerebral cortex (Ross and Tabrizi, 2011).

Clinically, there is a triad of motor, cognitive, and psychiatric problems observed in HD. The motor symptoms usually begin with chorea, muscle stiffness, dystonia, coordination difficulties, and dysarthria. Other possible motor symptoms that could develop later include bradykinesia, gait problems, and dysphagia (Bates et al., 2015; Walker, 2007). Cognition problems may affect executive functions such as planning and attention before progressing to memory loss and poor decision-making skills (Ross et al., 2014). The psychiatric problems include depression, anxiety, irritability, apathy, and sometimes even psychosis and obsessive–compulsive disorders. Suicide is an important consideration for patients with HD (Walker, 2007; Zuccato and Cattaneo, 2007).

Symptoms of juvenile HD (disease onset before the age of 20) frequently present themselves through a unique set of features, comprising of stiffness, clumsiness, seizures, learning difficulties, less chorea, and more rigidity (Quigley, 2017). Disease course tends to be relentlessly progressive, usually ending up with full dependency and death of the patient in 15–20 years from disease onset. According to a systematic review and meta-analysis of 33 research articles published in 2010–2022, global prevalence of HD is estimated at approximately 4.88 per 100,000 (Medina et al., 2022). However, there are significant variations depending on the geographical region. Prevalence in Asia is considerably lower (about 0.52 per 100,000), although higher figures were recorded in regions like Pakistan and India (Punjab, Gujarat) (Papanna et al., 2022, 2024). Latin American countries (Brazil, Peru, Mexico) register higher prevalence rates (0.64–54 per 100,000), with local pockets of cases recorded (Medina Escobar et al., 2024). In India, the latest report in 2024 estimates prevalence at around 7 per 100,000 (Research Nester, 2024).

Currently, there are no curative measures available for treating HD. The treatment measures available are largely symptomatic and focused on improving the quality of life. Tetrabenazine and Deutetrabenazine that reduce dopaminergic signaling have been FDA-approved for the treatment of chorea symptoms of HD but can be related to some side effects such as depression and Parkinsonism (Bachoud-Lévi et al., 2019). Antipsychotic drugs like risperidone and olanzapine, SSRIs like sertraline, and mood stabilizers like valproate are often used for treating psychiatric symptoms (Frank, 2014; Tabrizi et al., 2019). Physical, occupational, and speech therapy plays an important role in maintaining function and communication skills of the patient as much as possible.

However, these approaches fail to target the primary neurodegeneration problem. The heterogeneity of this condition as well as the challenges of penetrating the blood-brain barrier (BBB) with medications emphasizes the need for the development of therapeutic strategies targeting the core of the disease process. In the context of this approach, natural substances (phytochemicals) gain recognition because of their neuroprotective, antioxidant, and anti-inflammatory effects. Among them, curcumin (turmeric), resveratrol (grapes), and epigallocatechin gallate (EGCG, green tea) have shown promise in decreasing oxidative stress, regulating apoptosis, and blocking protein aggregation processes characteristic for HD (Chongtham and Agrawal, 2016; Ehrnhoefer et al., 2006; Hickey et al., 2012; Vidoni et al., 2018). Sesame seeds (*Sesamum indicum* L.) are Pedaliaceae family members that have been cultivated from ancient times because of their nutritional and medicinal value (Alves et al., 2023). Sesame seeds are known as “til” in Hindi language, “ellu” in Tamil language, and “zhī má” in Chinese language. The plant is extensively cultivated throughout tropical and subtropical areas, where its production is significant in India, China, Myanmar, Sudan, Nigeria, and Ethiopia (Dossou et al., 2023; Wei et al., 2022). Sesame seeds contain a variety of lignans like sesamin, sesamolin, and sesaminol, which make the plant a good source of antioxidants, anti-inflammatory, and neuroprotective agents (Andargie et al., 2021).

Based on their established roles in Huntington’s diseases pathogenesis, Huntingtin Exon 1 HTT-17Q, Caspases-6, PDE10A2, and SIRT1 were selected as therapeutic targets. HTT-17Q is involved in mutant huntingtin aggregation, Caspases-6 mediates proteolytic cleavage of huntingtin, PDE10A2 regulates striatal signaling, and SIRT 1 play a function in mitochondrial function and neuronal survival. HTT-17Q represents the wild-type form of the huntingtin protein’s N-terminal fragment with 17 glutamine residues. It serves as a reference for identifying disease-related conformational changes caused by polyQ expansions. As initial aggregation begins in Exon 1, targeting this region can help prevent toxic protein misfolding and aggregation (Cho, 2025; Guo et al., 2018). Caspase-6 is implicated in the cleavage of mutant huntingtin at residue 586, generating toxic fragments that contribute to early neuronal death. Inhibition of Caspase-6 has been shown to delay HD progression in preclinical models (Aharony et al., 2015; Ehrnhoefer et al., 2019; Graham et al., 2006). PDE10A is highly expressed in the striatum and regulates intracellular signalling via cAMP and cGMP. In HD, its inhibition restores cAMP levels, improves motor function, and reduces neuroinflammation (Layton et al., 2023; Raheem et al., 2016; Verhoest et al., 2009). Sirtuin 1 (SIRT1) is a NAD^+^-dependent deacetylase involved in regulating mitochondrial function, oxidative stress, and transcription. SIRT1 inhibition has been shown to reduce mHTT toxicity and promote neuronal survival (Duan, 2013). Following post-docking analysis, which included evaluation of docking scores, interaction types (e.g., hydrogen bonds, hydrophobic and π-π interactions), number of hydrogen bonds, and binding pocket characteristics, the top-ranked protein-ligand complexes were selected for MD simulations and binding free energy calculations. These analyses were performed to gain deeper insight into the stability, conformational dynamics, and binding strength of the complexes in a simulated biological environment.

The present study aimed to identify potential multi-target phytochemicals from *S. indicum* for Huntington’s diseases using an integrated experimental and computational approach. The methanolic seed extract was subjected to LC-MS/MS based phytochemical profiling, followed by ADMET screening to evaluate drug-likeness and safety. The selected compounds were subsequently assessed against key Huntington’s diseases associated protein targets, including HTT-17Q, Caspases-6, PDE10A2, and SIRT1, using molecular docking, molecular dynamics simulations and MMBPSA binding free energy analysis to identify promising lead compound.

## 2 Materials and methods

### 2.1 Preparation of sesame seeds extract

Sesame seeds were sourced from HW Wellness Solutions Private Limited. Extraction was carried out by cold maceration. One hundred grams of sesame seeds were pounded into a fine powder and 300 mL of methanol was added. The mixture was left to macerate for 7 days with intermittent shaking. After this period, the extract was filtered three times with Whatman No. 1 filter paper to obtain a clear extract. The filtrate was concentrated using a rotary evaporator at 40 °C and 70 rpm under decreased pressure. The yield of the obtained crude methanolic extract was calculated by weighing and then stored at 4 °C in an airtight container for future experimental study.

### 2.2 Identification and selection of chemical constituents

The crude methanolic extract of sesame seeds (*S. indicum*) was analysed for phytochemical profiling through liquid chromatography-mass-mass spectrometry (LC-MS/MS). The dried extract was dissolved in methanol prior to analysis. For the optimization of the LC method, the parameters that were varied included pressure from 0 to 1,034 bar, flow rate of 0.3 mL/min, run time of 35 min, and column compartment temperature of 40 °C. The mobile phases included solvent A (0.1% formic acid in water), solvent B (methanol), and solvent C (acetonitrile). For the mass spectrometric analysis, the high-resolution Q-Exactive Plus Orbitrap LC-MS/MS (Thermo Fisher Scientific Pvt. Ltd.) was employed in the mode of ESI positive/negative ionization. The scan range was set from 70 to 1,000 m/z. Injection of the sample was made using Hamilton syringe with a flow rate of 3 μL/min, internal diameter of 2.3 mm, and total volume of 250 μL. Spectra obtained in this way were analyzed by means of comparison with the natural products libraries. Phytoconstituents were selected based on the intensity of the peaks and high mass accuracy; only compounds with the identification confidence >95% were taken into consideration for interpretation of the results.

### 2.3 Pharmacokinetic and pharmacodynamic predictions

Pharmacokinetic and pharmacodynamic parameter evaluation is crucial for preventing drug failure in subsequent clinical trial steps. These shortlisted molecules were compiled into a CSV file, which included several pieces of information, such as the name of the compound, PubChem ID, and its SMILES annotation. These compounds were passed through Absorption, distribution, metabolism, and excretion (ADME) prediction using the SwissADME web tool (Daina et al., 2017). At this step, filters such as High GI absorption, BBB (Yes), Lipinski violations (0), and PAINS alerts (0) were used to critically evaluate the selected compounds. The compounds that followed the above parameters were subjected to the ProTox 3.0 tool for toxicity evaluation (Banerjee et al., 2024). Filters used in toxicity evaluation were Hepatotoxicity, neurotoxicity, carcinogenicity, and cytotoxicity.

### 2.4 Compound retrieval and ligand library preparation

A total of eight compounds passed ADME and toxicity assessment, which were retrieved through the PubChem database (https://pubchem.ncbi.nlm.nih.gov/) in SDF format. The selected compounds were further prepared using the MMFF94 force field for 1,000 steps in the Open Babel Linux GUI (O’Boyle et al., 2011). The prepared compounds were saved in Autodock-compatible pdbqt format.

### 2.5 Target selections and retrieval

HD is a rare, inherited neurodegenerative disorder caused by a CAG repeat expansion in the Huntingtin gene, leading to the production of a toxic protein that progressively damages brain cells (Hughes et al., 2014; Zheng and Diamond, 2012). This condition is characterised by a range of symptoms, including chorea, which involves involuntary, jerky movements, cognitive decline, and psychiatric disturbances (Bobori, 2015). The severity and onset of symptoms are influenced by the length of the CAG repeat; longer expansions are associated with earlier onset (Zdzienicka et al., 2002). Presymptomatic testing is available for individuals with a history of HD, allowing them to understand their genetic risk before symptoms appear, which can aid in family planning and genetic counselling. After studying the pathology of HD, we selected the disease-related protein targets. The selected protein targets were (a) Huntingtin Exon 1 (HTT)-17Q variant (PT1) (PDB ID: 4CBY), (b) huCaspase-6 in complex with inhibitor 3a (PT2) (PDB ID: 8EG5), (c) Crystal structure of PDE10A2 with fragment ZT464 (PT3) (PDB ID: 4LM3), and (d) Sirtuin1 (SIRT1) (PT4) (PDB ID: 4I5I). Fortunately, all the selected targets exhibited co-crystallised ligand molecules, which helped us to determine the experimentally proven binding pocket for each protein. The co-crystallised ligands respective to each protein were used as a reference for further comparison in this study. Also, only A chain was considered in all the selected targets because they were homodimers in nature, except huCaspase-6 in complex with inhibitor 3a (PT2) to reduce the computational cost. Two chains were heterodimers in nature for PT3. Therefore, Chain A and Chain B were both considered for PT2. We have also confirmed the biological conformational existence of each of the selected proteins.

### 2.6 Molecular docking using AutoDock Vina

The retrieved targets were prepared using the AutoDock 4.2 tool (Morris et al., 2009). To do so, for each protein target, removal of water molecules and heteroatoms, along with the addition of polar hydrogen and Gasteiger charges, was executed. The prepared proteins were saved in AutDock-compatible pdbqt format. A site-specific grid box for each target protein was created at the experimentally proven binding site, which was earlier occupied by the co-crystallised ligand (reference compound). Since it resembled the experimentally proven binding pocket of each protein. The detailed information on the grid box dimension is mentioned in Table 1. The self-docking was performed, and the RMSD values were calculated to validate the docking procedure. Lastly, AutoDock Vina was used to perform the molecular docking process against targets PT1, PT2, PT3, and PT4 for the eight previously produced compounds and the corresponding co-crystallized ligands (Jaghoori et al., 2016). For additional research, each protein-ligand complex was thoroughly examined.

**TABLE 1:** Selected protein targets with their Grid size used in molecular docking.

S. no.	Target	Name	PDB ID	Resolution (in Å)	Experimental method	Centre x × center y × center z	Size x × size y × size z	Reference
1	PT1	Huntingtin Exon 1 (HTT)-17Q variant	4CBY	2.72	X-ray diffraction	24.747 × 1.206 × 36.715	48 × 48 × 62	Bürli et al. (2013)
2	PT2	huCaspase-6 in complex with inhibitor 3a	8EG5	2.14	X-ray diffraction	31.912 × −11.699 × −38.482	56 × 74 × 68	Van Horn et al. (2023)
3	PT3	Crystal structure of PDE10A2 with fragment ZT464	4LM3	1.49	X-ray diffraction	6.274 × 11.203 × 42.899	52 × 48 × 56	Recht et al. (2014)
4	PT4	Sirtuin1 (SIRT1)	4I5I	2.50	X-ray diffraction	35.001 × −16.391 × 26.347	76 × 90 × 57	Zhao et al. (2013)

### 2.7 Post-docking analysis

Discovery Studio Visualizer (DSV) (https://discover.3ds.com/discovery-studio-visualizer-download) was used to further analyse each protein-ligand complex for docking score, bond types, and binding pocket size. The hit compounds were pursued for molecular dynamics simulation studies since they were shown to act on many targets and produce superior results than the corresponding co-crystallized ligand (reference molecule).

### 2.8 Molecular dynamics simulations

Generally, in the molecular docking step, the protein is made to be rigid and the ligand to be flexible, whereas to mimic the actual physiology, both should be flexible. To meet such requirements, Molecular dynamics simulations (MDS) studies are frequently considered in several studies (Sharma and Muniyan, 2024). The selected protein-ligand complex was taken for MDS using the GROMACS 24.1 tool (Abraham et al., 2015) in pre-built workflows in ScientiFlow (https://scientiflow.com/). The ligand topology was obtained using the SwissParam web tool (Zoete et al., 2011). On the other hand, protein topology was created using the “gmx pdb2gmx” GROMACS utility along with the charmm36 force field. The CHARMM36 force field was selected for protein parameterization owing to its reliable ability to recreate the physical behaviour of proteins. To generate ligand topologies, we used SwissParam, a tool that generates CHARMM-compatible ligand parameters on the basis of the principles of the CHARMM General Force Field (CGenFF), thus allowing complete integration with CHARMM36 in GROMACS. This methodology has been successfully used in many previous studies related to the interactions between proteins and ligands (Bugnon et al., 2023). A 1 nm triclinic box and the TIP3 water molecule were utilized for additional protein topological execution. Sodium and chlorine ions were added as needed to neutralize the protein-ligand system. Additionally, throughout the NVT and NPT stages, position restraint and an energy minimization step for 50,000 steps were carried out, stabilizing the temperature at 300K and pressure at 1 bar. For every protein-ligand combination, a 200 ns (20,000 frame) simulation was run. Afterward, trajectory analysis was performed using metrics including root mean square deviation (RMSD), root mean square fluctuation (RMSF), solvent accessible surface area (SASA), radius of gyration (Rg), and H-bond.

### 2.9 Binding free energy calculations

Using the gmx_MMPBSA tool (Valdés-Tresanco et al., 2021), the chosen protein-ligand complex was also considered for the calculation of binding free energy (Molecular Mechanics Poisson-Boltzmann Surface Area (MM-PBSA)). gmx_MMPBSA used MMPBSA. py by AMBER to calculate the binding free energy using GROMACS-generated files (Miller et al., 2012). As a stable region during the simulation procedure, the final 10 ns (1,000 frames) of the corresponding MDS trajectory were chosen to compute binding free energy (ΔGbinding). The complex’s stability was examined and contrasted appropriately.

## 3 Results

### 3.1 Extractions

The cold maceration method was used to extract the sesame seeds using methanol. The yield of methanolic extract was determined to be 10.38 g/kg w/w.

### 3.2 Compound identification

LC–MS/MS was used to analyse the acquired fraction in both positive and negative electrospray ionization (ESI) modes. While 74 compounds were found in the positive ionization mode, a total of 91 compounds were tentatively identified in the negative ionization mode. As seen in Table 2, 86 of these compounds showed high-intensity signals and were recognized with a confidence level more than 95%. Supplementary Materials S2–S5 contain representative chromatograms produced in both positive and negative ionization regimes.

**TABLE 2:** Tentative identification of selected phytochemicals with their molecular formula, protonated molecular ion (m/z), and approximate retention time (RT) obtained by LC–MS analysis.

Compounds	RT	Mass	Formula	DB Diff (ppm)	Hits (DB)
Isosorbide dinitrate	1.235	236.027	C6H8N2O8	2.71	2
Retronecine	1.578	155.0945	C8H13NO2	0.9	3
Nicotinamide N-oxide	1.612	138.0421	C8H13NO2	0.9	3
Hypoglycin	1.745	141.0785	C7H11NO2	3.43	4
Pirbuterol	1.895	240.1455	C12H20N2O3	7.76	1
Marimastat	2.215	331.2094	C15H29N3O5	3.99	1
6-Hydroxynicotinic acid	2.466	139.0268	C6H5NO3	0.99	6
Domeperidone	3.171	425.1644	C22H24ClN5O2	−5.93	1
Metharbital	3.56	198.1012	C9H14 N2O3	−3.78	5
Prolyl-Tyrosine	3.984	278.124	C14H18N2O4	9.54	5
Simeconazole	4.583	293.1356	C14H20FN3OSi	1.27	5
Euglobal-Ia1	4.644	386.2067	C23H30O5	6.89	10
Pymetrozine	4.672	217.0954	C10H11N5O	4.43	2
Indoleacrylic acid	4.726	187.0631	C11H9NO2	1.05	10
L-Tryptophan	4.727	204.0891	C11H12N2O2	3.61	9
Thiorphan	4.929	253.0771	C12H15NO3S	0.81	7
D-Tryptophan	4.932	204.0885	C11H12N2O2	6.62	8
Dizocilpine	4.997	221.1215	C16H15N	−4.62	1
Maculosin	5.209	260.1153	C14H16N2O3	3.12	5
Niazirin	5.911	279.1086	C14H17NO5	7.32	4
Harzianopyridone	6.373	281.1258	C14H19NO5	1.79	3
Cimifugin	6.583	306.1101	C16H18O6	−0.11	4
Gentianamine	7.962	205.0728	C11H11NO3	5.16	10
Tocainide	8.27	192.1257	C11H16N2O	2.72	1
[4]-Gingerdiol 3,5-diacetate	8.542	352.1878	C19H28O6	2.13	10
Urapidil	8.552	387.2241	C20H29N5O3	7.5	6
Pitavastatin	8.565	421.1696	C25H24FNO4	−1.46	2
Licoagrodione	9.14	356.1229	C20H20O6	8.72	10
Prosolanapyrone II	9.051	304.1669	C18H24O4	1.88	10
Phenylacetic acid	8.837	136.0521	C8H8O2	2.09	10
N-heptanoyl-homoserine lactone	9.814	213.1367	C11H19NO3	−0.78	3
Chryso-obtusin	9.949	358.1015	C19H18O7	10.61	7
Garcinia lactone dibutyl ester	10.458	302.1347	C14H22O7	5.99	6
3,3'-Dihydroxy-4',5,7-trimethoxyflavan	11.165	332.1253	C18H20O6	2.18	10
3,4',5,6,8-Pentamethoxyflavone	11.174	372.1174	C20H20O7	9.45	5
C16 Sphinganine	12.897	273.2646	C16H35NO2	8.1	1
Sesamolin	13.154	370.1016	C20H18O7	9.74	6
Ricinoleic acid	14.86	298.2488	C18H34O3	6.54	6
Monomenthyl succinate	19.719	256.1672	C14H24O4	0.86	6
Sevoflurane	1.617	200.0065	C4H3F7O	3.38	10
Tuliposide A	1.783	278.0982	C11H18O8	7.12	9
Diamidafos	1.799	175.083	C8H13N2O2P	2.8	5
(±)-alpha-Narcotine	1.888	413.1517	C22H23NO7	−10.19	3
Calystegine B5	1.938	175.083	C7H13NO4	8.12	10
Zonisamide	2.113	212.0234	C8H8N2O3S	10.24	1
Ranunculin	2.304	276.0822	C11H16O8	8.28	3
Quinic acid	3.28	192.0624	C7H12O6	5.11	6
Rotenonone	2.945	406.106	C23H18O7	−1.79	10
Sporidesmin	4.434	473.0444	C18H20ClN3O6S2	7.98	2
Glucoalyssin	4.467	451.0634	C13H25NO10S3	1.45	2
Vulgaxanthin-II	4.783	340.0899	C14H16N2O8	2.24	10
Niazirin	5.969	279.1088	C14H17NO5	6.63	3
Flumioxazin	7.361	196.0718	C19H15FN2O4	−11.69	10
Xanthoxylin	7.396	354.1057	C10H12O4	0.99	10
S-Nitroso-L-glutathione	7.614	336.0739	C10H16N4O7S	0.09	10
Eupatundin	7.685	376.1496	C20H24O7	6.88	10
Mytilin A	7.745	332.1239	C13H20N2O8	−5.75	10
N-[(5-Hydroxy-2-pyridinyl)methyl]adenosine	7.815	374.134	C16H18N6O5	−3.7	5
Gibberellin A60	7.961	358.1398	C19H24O6	6.26	10
Elephantin	8.085	584.2984	C20H22O7	3.25	10
Arginyl-Asparagine	8.142	346.1756	C10H20N6O4	−3.01	6
Spenolimycin	8.337	288.1555	C15H26N2O7	8.82	7
Euphornin	8.492	358.104	C33H44O9	−4.69	10
2-Phenylaminoadenosine	8.561	348.1551	C16H18N6O4	−3.7	10
16alpha-Bromo-17beta-estradiol	8.764	374.1353	C18H23BrO2	−1.03	5
Thyrotropin-releasing hormone	8.826	362.1718	C16H22N6O4	−4.36	10
Peroxyacetic acid uroporphyrin III	9.414	568.3037	C40H38N4O17	−6.91	10
5-Oxoavermectin ''1b'' aglycone	9.502	396.2164	C33H44O8	0.23	6
Scorzoside	9.756	408.1681	C21H30O8	−1.78	7
Isopetasoside	10.11	396.216	C21H32O7	3.52	10
Beclomethasone	9.901	446.3681	C22H29ClO5	0.23	5
Aflatoxin ExB2	9.94	318.174	C19H18O7	5.43	10
Pteroic acid	10.279	312.0977	C14H12N6O3	0.51	2
Perindopril	14.868	368.2296	C19H32N2O5	4.24	1
Rhazidigenine Nb-oxide	14.835	314.1943	C19H26N2O2	16.48	10
Valdiate	20.238	310.1793	C17H26O5	−3.7	1
3-Hydroxypropyl oleate	15.237	341.305	C21H40O3	−6.97	10
9,12 - Octadecadienoyl chloride	16.457	299.2142	C18H31ClO	8.82	10
Z-4- Nonadecen-1-ol acetate	16.753	325.3101	C21H40O2	2.24	3
Brassidic Acid	18.542	339.3258	C22H42O2	0.51	5
E-11(12-Cyclopropyl) dodecen-1-ol	14.854	225.2213	C15H28O	−4.36	6
9-octadecyne	16.887	251.2733	C18H34	−1.79	10
Gossyplure	18.426	283.2632	C18H34O2	−10.19	1
Methyl 2,6,10- trimethyltridecanoate	17.886	271.2632	C17H34O2	−1.46	1
Sesamin	12.223	354.11	C20H18O6	−4.2	10
Ricinoleic acid	14.86	298.2488	C18 H34 O3	6.54	6

### 3.3 ADMET predictions

Using reliable technologies, 86 different compounds were collected from the HR-LCMS-QTOF study and shortlisted for the first round of ADMET screening. Fourteen of the eighty-six compounds showed considerable promise for oral bioavailability due to their high gastrointestinal absorption. Furthermore, those 14 drugs demonstrated good BBB permeability, suggesting the possibility of reaching HD-related CNS targets. Interestingly, these 14 compounds also showed no violations for Ghose, Veber, Egan, Muegge, Lipinski’s Rule of Five, and PAINS filters, indicating their drug-like properties. Additionally, eight compounds were determined to be fully harmless for hepatotoxicity, neurotoxicity, carcinogenicity, and cytotoxicity after being exposed to the ProTox 3.0 web server. The predictive potential of these eight compounds for targets relevant to Huntington’s disease was further investigated. The complete details regarding ADMET for the eight shortlisted compounds have been mentioned in Supplementary Tables S1, S2.

### 3.4 Molecular docking

Using the AutoDock Vina tool, a stiff protein and flexible ligand-based molecular docking was carried out for the eight chosen compounds against PT1, PT2, PT3, and PT4. Table 1 already has information on the binding site and grid size. The RMSD values of the docked complexes and the co-crystallized ligands were determined by superimposition to validate the docking procedure in Pymol software. The estimated RMSD values were PT1 (0.363 Å), PT2 (1.038 Å), PT3 (0.00 Å), and PT4 (3.041 Å). The superimposed RMSD values were near or less than 3Å, which lies within the range of self-docking or docking validation protocol (Bhusal et al., 2024). At this stage, the range of the greatest and minimum docking scores was found to be between −4.6 and −10.3 kcal/mol. To facilitate comparability, post-docking analysis was performed on each protein-ligand complex to verify the kinds and quantity of bonds present in each complex. To compare the test molecule to the corresponding protein target, we used the co-crystallized ligand as a reference compound.

### 3.5 Post-docking analysis

Supplementary Table S3 represents the entire view for eight filtered compounds docked against PT1, PT2, PT3, and PT4. We can see Supplementary Table S3, on PT1 reference compound showed the docking score of −10.3 kcal/mol upon formation H Bond with HIS842, VD with residues ASP759, GLY811, PHE812, PRO800, ARG681, GLU677, HIS976, PRO676, GLY974, GLU973, LEU943, GLY975, HIS803, ASP840, and ASP934, along with some other bonds at PHE871, HIS802. Unfortunately, no test compounds showed a lower docking score on PT1. A similar pattern was observed on PT2, where the reference compound had a docking score of −9.4 kcal/mol forming different bonds such as H Bond at ARG220, SER218, SER226, ARG64, and CYS264, VD at GLY66, THR67, GLN161, HIS219, LYS265, ASP266, TYR217, HIS121 and some other bonds with ASP70, GLY225, ARG65, CYS163, and PRO62 residues. On PT2, none of the test compounds performed better than the reference compound.

The trend was dissimilar against PT3. The reference compound had a docking score of −6.7 kcal/mol with interactions with residues such as: VD: Leu635, Val768, Thr685, Thr688, Ala689, Phe696, Gln726, Tyr730, Other bonds: LEU675, ILE692, and PHE729. The docking score of the reference compound was comparatively higher, and no hydrogen bond formation was observed. Four test compounds showed a docking score lesser than the reference compounds against the PT3 protein target, and these were 16 alpha-Bromo-17beta-estradiol (−9.3 kcal/mol), 3,4',5,6,8-Pentamethoxyflavone (−8 kcal/mol), Prosolanapyrone II (−7.5 kcal/mol), and Valdiate (−7.3 kcal/mol).

Against PT4, the reference compound exhibited a docking score of −8.3 kcal/mol upon interacting with key residues such as: H bond: HIS363, VD: ARG274, GL2N345, ASN346, PHE273, VAL412, and some other bonds: ILE347, PHE297, ILE316, ILE411, VAL445, and PHE414. Interestingly, on PT4 as well, there was one test compound (Valdiate) showing a lower score against PT4, i.e., −8.9 kcal/mol upon interacting in the same binding as that of the reference compound. The key interactions of Valdiate on PT4 were found to be C-H Bond: GLN345, VD: SER265, ASN346, ILE316, VAL412, TYR280, VAL445, HIS363, ASP272, ALA262, PRO271, ILE270 ASP348, and some other bonds: ILE347, ILE411, PHE297, PHE273, PHE413, PHE414.

**FIGURE 1:**
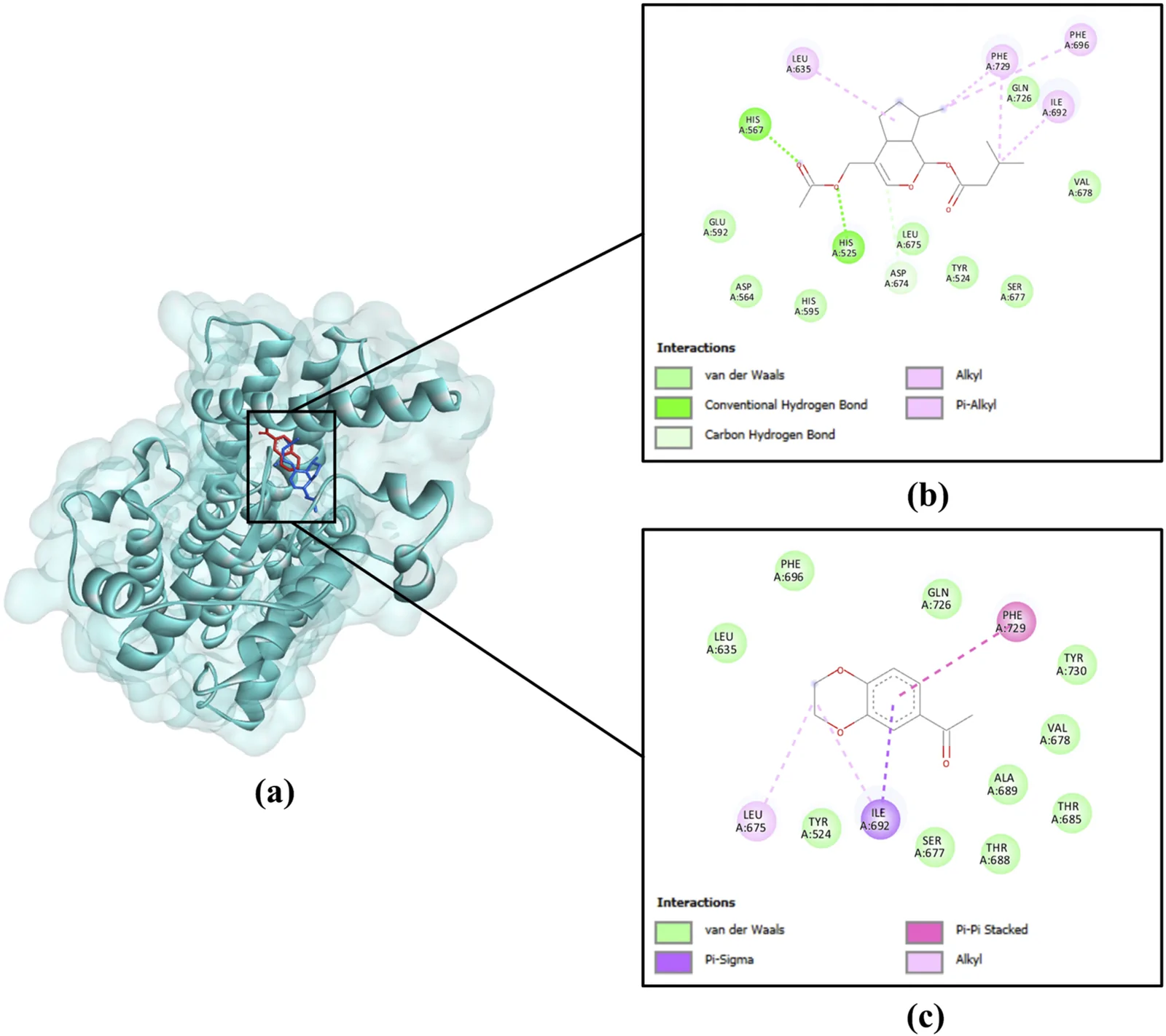
**(a)** Superimposition of test compound (blue) with reference compound (red) in the binding pocket of PT3 (cyan), **(b)** 2D representation of PT3_Test, and **(c)** 2D representation of PT3_Reference.

**FIGURE 2:**
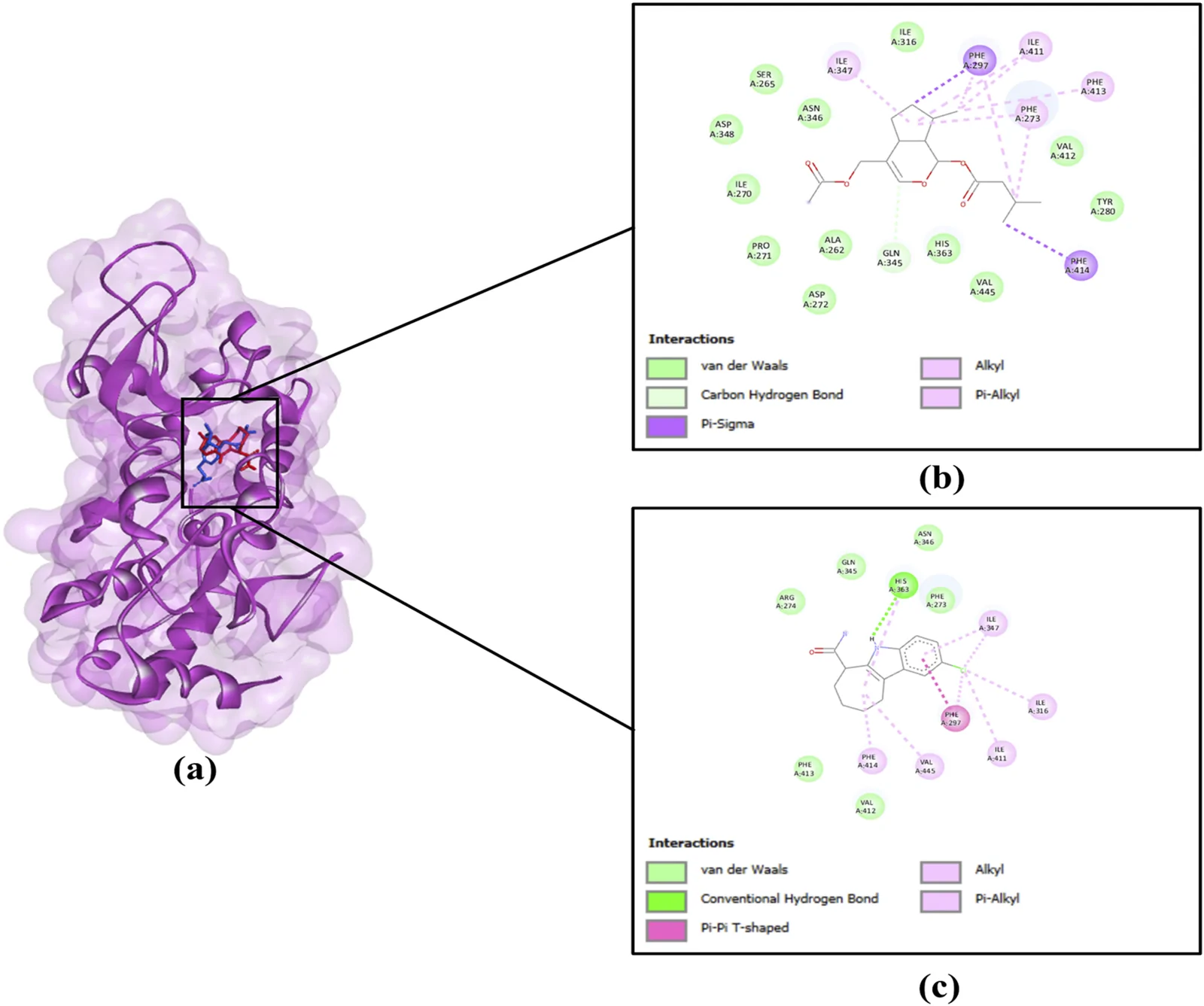
**(a)** Superimposition of test compound (blue) with reference compound (red) in the binding pocket of PT4 (purple), **(b)** 2D representation of PT4_Test, and **(c)** 2D representation of PT4_Reference.

Upon comparing all possible protein-ligand complexes with the reference compounds, it can be concluded that not a single test compound performed better than the reference compound against PT1 and PT2. Whereas four tests, as mentioned before, on PT3 and one on PT4 performed better than their respective reference compounds. From the above post-docking analysis, we observed that the compound Valdiate (PuChem ID: 129715809) performed better on PT3 and PT4 with good binding energies, binding pocket, and types of interaction. Our selection of Valdiate was based on the primary objective of this study: identifying a multi-target candidate for Huntington’s disease, rather than selecting the top-scoring ligand against a single protein target. Therefore, we selected the compound that performed better on more than one target. Notably, compounds such as 16 alpha-Bromo-17beta-estradiol and 3,4',5,6,8-Pentamethoxyflavone exhibited lower docking scores than Valdiate against PT3; they did not show comparable performance against PT4. In contrast, Valdiate demonstrated favorable binding to both PT3 (−7.3 kcal/mol) and PT4 (−8.9 kcal/mol), occupied the same binding pocket as the reference ligand, and formed relevant interactions with key residues on both targets. Therefore, Valdiate docked with PT3 and PT4, along with their respective reference compound, were further considered for MDS. This step would enable us to get deeper insights into the stability of each protein-ligand complex. The detailed representation of Vadiate (test) compound docked with PT3 (PT3_Test), Reference compound docked with PT3 (PT3_Reference), Vadiate (test) compound docked with PT4 (PT4_Test), and Reference compound docked with PT4 (PT4_Reference) has been shown in Figures 1, 2.

### 3.6 Molecular dynamics simulations (MDS) studies

Additionally, MDS investigations were carried out to examine the protein-ligand complex’s stability in a dynamic setting. Using GROMACS 24.1, four protein ligand systems-PT3_Test, PT3_Reference, PT4_Test, and PT4_Reference-were taken into consideration for MDS. For every protein-ligand combination, the MDS trajectories were run for 200 ns. Several analyses were performed, including Protein-RMSD, Ligand-RMSD, RMSF, RG, SASA, and the quantity of H-bond forms.

### 3.7 Protein-RMSD trajectories analysis

**FIGURE 3:**
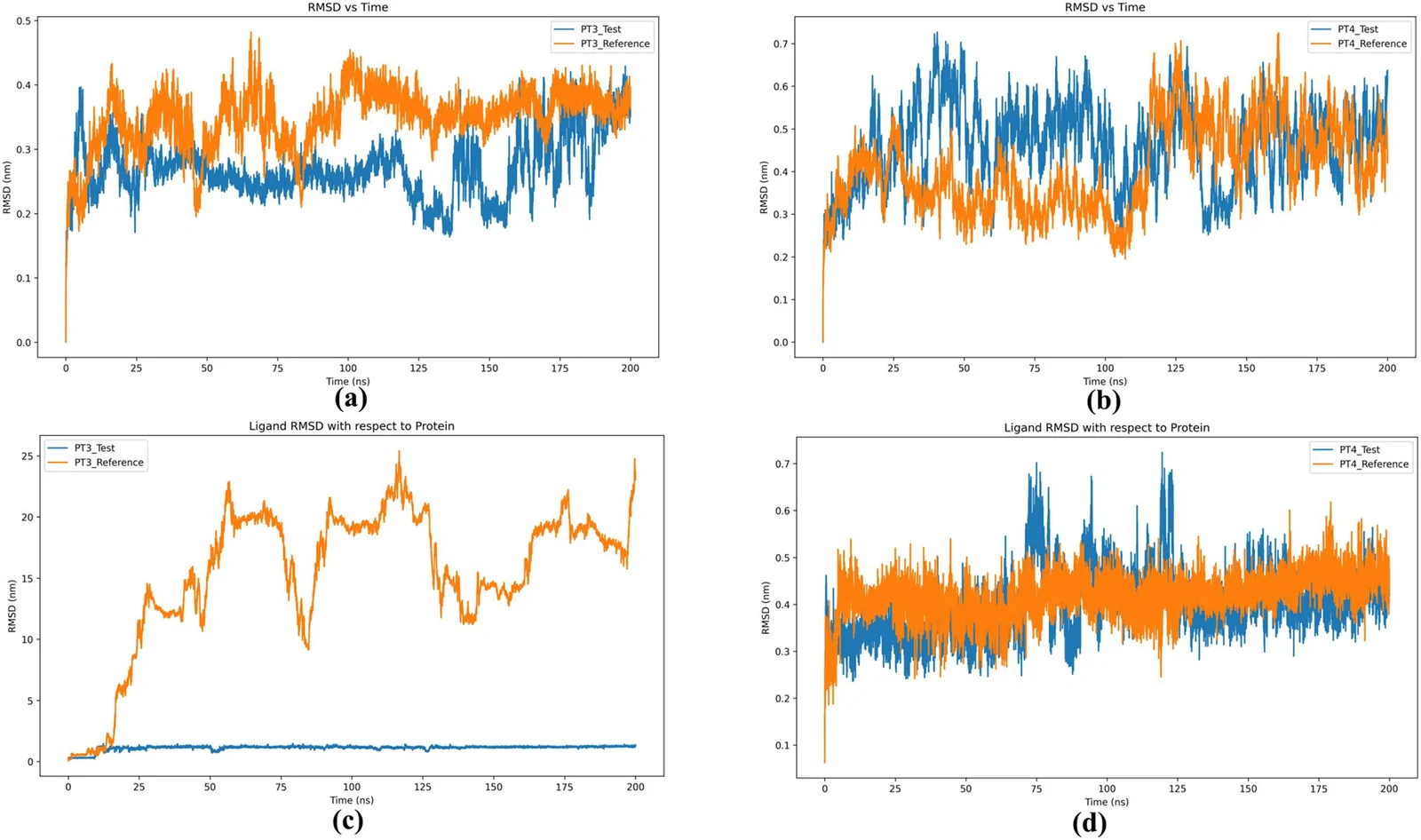
Representation of **(a)** P-RSMD for PT3_Test (blue) and PT3_Reference (orange), **(b)** P-RSMD for PT4_Test (blue) and PT4_Reference (orange), **(c)** L-RSMD for PT3_Test (blue) and PT3_Reference (orange), and **(d)** L-RSMD for PT4_Test (blue) and PT4_Reference (orange).

The root mean square deviation of the protein’s backbone from its initial condition is examined using Protein-Root Mean Square Deviation (P-RMSD) analysis. In general, the protein-ligand combination is more stable when the RMSD value is lower. In Figure 3a, the equilibration time (initial time taken by every trajectory to become stable) for PT3_Test and PT3_Reference was 4.6 ns and 7 ns, respectively. The RMSD range for both trajectories was from 0.15 to 0.48 nm. The major and minor fluctuations in PT3_Test trajectory were found at 4.6 ns and 129, ns, 139 ns, 152 ns, and 160 ns, respectively, whereas in PT3_Reference they were observed at 15 ns, 35 ns, 45 ns, 67 ns, 83 ns, 100 ns, 30 ns, and 170 ns, respectively. Upon comparison of both trajectories, it could be seen that PT3_Test was stable in the region (48–107) ns and PT3_Reference in the region (129–200) ns, with some minute fluctuations. Overall average RMSD values for PT3_Test and PT3_Reference were (0.269 ± 0.045) nm and (0.347 ± 0.046) nm, respectively (Table 3). Based on the trends in trajectory and average P-RMSD value, it could be said that the test compound was more stable than the reference compound on the PT3 protein target.

**TABLE 3:** Average values of P-RMSD (nm), L-RMSD (nm), RMSF (nm), RG (nm), SASA (nm^2^), Number H-bonds, and Total Binding free energy calculations.

Analysis	PT3_test	PT3_reference	PT4_test	PT4_reference
P-RMSD (nm)	0.269 ± 0.045	0.347 ± 0.046	0.460 ± 0.093	0.407 ± 0.099
L-RMSD (nm)	1.124 ± 0.204	15.311 ± 5.698	0.398 ± 0.069	0.417 ± 0.045
RMSF (nm)	0.123 ± 0.147	0.136 ± 0.122	0.172 ± 0.189	0.189 ± 0.206
RG (nm)	2.039 ± 0.013	2.067 ± 0.016	2.085 ± 0.026	2.061 ± 0.040
SASA (nm^2^)	168.13 ± 3.74	172.19 ± 4.12	157.36 ± 2.95	154.99 ± 3.43
Number H-bonds	0–2	0–3	0–3	0–5
Total Binding free energy (kcal/mol)	−23.22 ± 2.45	0.00 ± 0.00	−28.87 ± 2.28	−19.80 ± 3.68

Similarly, Figure 3b illustrates that the P-RMSD values of the test and reference compounds on PT4 ranged from 0.1 nm to 0.7 nm. While minor fluctuations were seen more frequently, the largest fluctuations in PT4_Test were 40 ns, 59 ns, 105 ns, 128 ns, and 135 ns. After 155 ns, the trajectory remained quite constant until 200 ns. The PT4_Reference trajectory showed numerous minor fluctuations at 25 ns, 125 ns, and 162 ns, as well as a single major at 114 ns. From 28 ns to 116 ns and from 116 ns to 200 ns, the PT4_Reference trajectory remained steady. Analysing the trajectory graphs alone made it difficult to comment on the stability. Therefore, the average P-RMSD value was calculated as (0.460 ± 0.093) nm for PT4_Test and (0.407 ± 0.099) nm for PT4_Reference compound (Table 3). Upon comparison of these trajectories, the reference compound was comparatively stable on PT4 than the test compound.

### 3.8 Ligand-RMSD trajectories analysis

The ligand molecule’s departure from its original state over the course of the simulation is shown by the Ligand-Root Mean Square Deviation (L-RMSD). In Figure 3c, on PT3, the reference compound shows notable variations with multiple drastic peaks that extend up to 25 nm, while the test compound is quite stable, as seen by a stable plot throughout the simulation duration. The computed average L-RMSD values for PT3_Test (1.124 ± 0.204 nm) and PT3_Reference (15.311 ± 5.698 nm) likewise showed this phenomenon (Table 2). The ligand’s rotation or flipping within the binding pocket could be the cause of this high L-RMSD value. Even if it stays bound, this can still happen. Another possibility is that inadequate interactions between the ligand and protein residues caused the ligand to exit the binding pocket. Comparative stability between PT4_Test and PT4_Reference was seen in Figure 3d, with average L-RMSDs of 0.398 ± 0.069 nm and 0.417 ± 0.045 nm, respectively (Table 3).

#### 3.8.1 RMSF trajectories analysis

**FIGURE 4:**
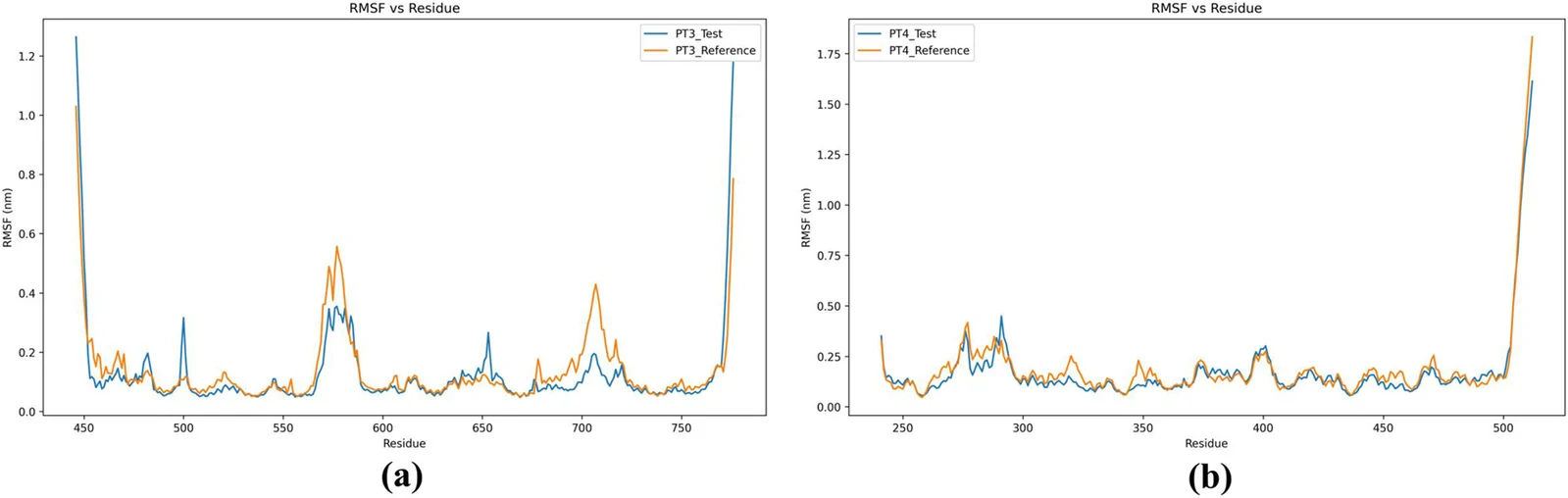
Representation of RMSF plots **(a)** PT3_Test (blue) and PT3_Reference (orange), and **(b)** PT4_Test (blue) and PT4_Reference (orange).

Each residue’s variation over the course of the simulation is displayed in the Root Mean Square Fluctuation (RMSF) graphic. A stable protein-ligand combination is typically associated with a lower RMSF value. The main changing residues on the PT3 target in Figure 4a are those between 560 and 580 and 675 and 725. PT3_Test and PT3_Reference have average RMSF values of (0.123 ± 0.147) nm and (0.136 ± 0.122) nm, respectively. Similarly, a fluctuating area was observed between 275 and 300 residues in Figure 4b. The average RMSF values of PT4_Test and PT4_Reference (0.172 ± 0.189 nm and 0.189 ± 0.206) nm (Table 3). There was no significant difference in the average RMSF values of the trajectories, however the test compound was marginally more stable than the reference compound on both PT3 and PT4 targets.

#### 3.8.2 RG trajectories analysis

**FIGURE 5:**
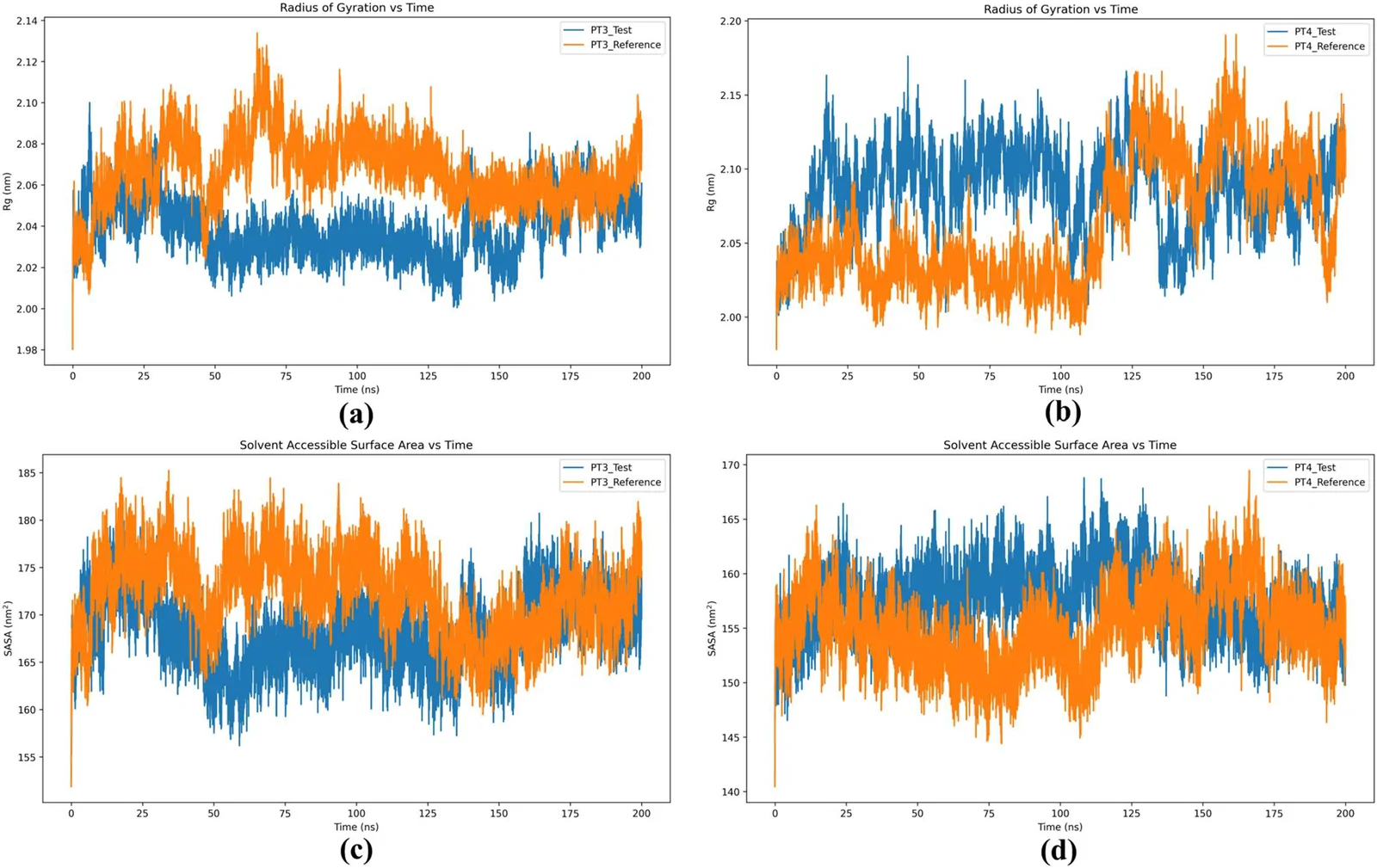
Representation of **(a)** RG plots for PT3_Test (blue) and PT3_Reference (orange), **(b)** RG plots for PT4_Test (blue) and PT4_Reference (orange), **(c)** SASA graph for PT3_Test (blue) and PT3_Reference (orange), and **(d)** SASA graph for PT4_Test (blue) and PT4_Reference (orange).

The radius of gyration (RG) defines compactness of protein. Generally, lower RG value suggests better compactness and more stability of the protein-ligand combination. Figures 5a,b show RMSF plots of the test and reference compounds on PT3 and PT4 targets, respectively. The range of RG values for all four trajectories ranged from 2.00 to 2.20 nm, i.e., within acceptable ranges. For PT3_Test, PT3_Reference, PT4_Test, and PT4_Reference, the average RG values were found to be 2.039 ± 0.013 nm, 2.067 ± 0.016 nm, 2.085 ± 0.026 nm, and 2.061 ± 0.040 nm, respectively (Table 3). The average RG values of the test were near enough to those of the reference drug on both the PT3 and PT4 targets, which prevented us from commenting on stability based on RG plots.

#### 3.8.3 SASA trajectories analysis

The Solvent Accessible Surface Area (SASA) represents the surface area of a protein structure that is accessible to solvent molecules. In general, a lower SASA value implies the stability of the protein-ligand system. The range of SASA values falls from 157 to 185 nm2 for PT3 and 145–168 nm2 for PT4, and both are within acceptable limits. The SASA values average for PT3_Test, PT3_Reference, PT4_Test, and PT4_Reference, were determined as (168.13 ± 3.74) nm2, (172.19 ± 4.12) nm2, (157.36 ± 2.95) nm2, and (154.99 ± 3.43) nm2, respectively (Table 3). Based on SASA values, it could be said that the test compound gained greater stability on PT3 but not on PT4 than the reference compound (Figures 5c,d).

#### 3.8.4 Number of H-bonds

The more hydrogen bonds contribute to a more stable protein-ligand complex, while certain other bonding, such as hydrophobic interactions, may be responsible for the stability in some rare circumstances. In Figures 5a,b, total (0–2), (0–3), (0–3), and (0–5) hydrogen bonds were found in PT3_Test, PT3_Reference, PT4_Test, and PT4_Reference, respectively. As stated previously, the other analysis reveals the stability of the test compound over the reference, however the number of H-bonds demonstrates that there is a fewer number of H-bond formations in the test compound. In place of that, there can be other hydrophobic interactions aiding the test chemical reach stability over the reference compound (Table 3).

#### 3.8.5 Binding free energy calculations

**FIGURE 6:**
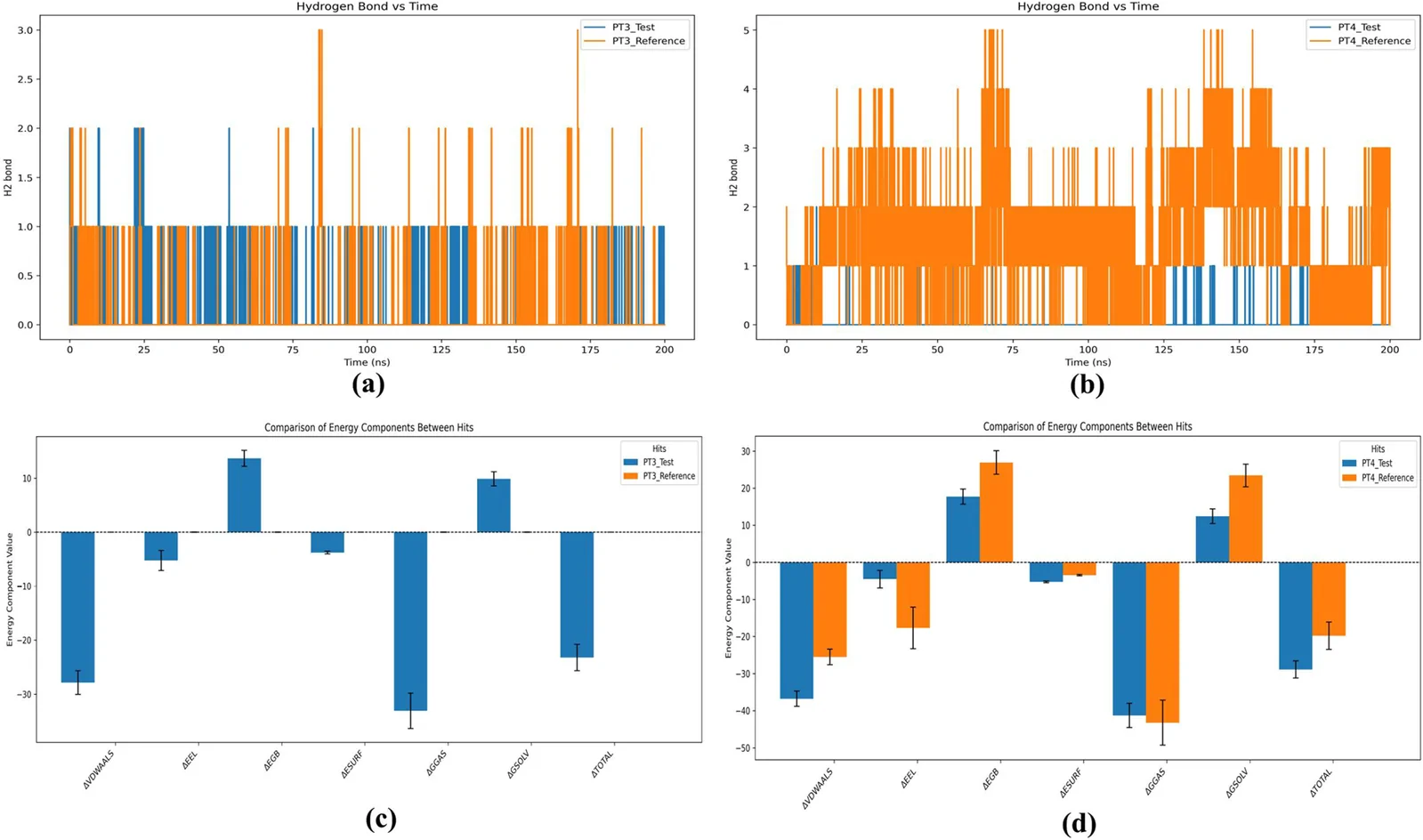
Representation of **(a)** H-bonds for PT3_Test (blue) and PT3_Reference (orange), **(b)** H-bonds for PT4_Test (blue) and PT4_Reference (orange), **(c)** binding free energy calculation for PT3_Test (blue) and PT3_Reference (orange), and **(d)** binding free energy calculation for PT4_Test (blue) and PT4_Reference (orange).

A more recent and sophisticated investigation is gmx_MMPBSA, which further verifies the protein-ligand complex’ stability. Total binding free energy was calculated for 1,000 frames for the four protein-ligand complexes that were chosen: PT3_Test, PT3_Reference, PT4_Test, and PT4_Reference. Stable protein-ligand complexes have lower negative total binding energies. From the output of MMPBSA, there was an aligned interpretation of L-RMSD and MMPBSA for the PT3_Reference complex. The value of the total binding energy was obtained as zero. This may be again due to insufficient stronger binding interactions to keep the reference ligand in the binding pocket of PT3. In contrast, PT3_Test attained a total binding energy of (−23.22 ± 2.45 kcal/mol) that marked its stability over PT3_Reference. The PT4_Test and PT4_Reference complexes attained a total binding energy of (−28.87 ± 2.28 kcal/mol) and (−19.80 ± 3.68 kcal/mol). This clearly defined the stability of PT4_Test over PT4_Reference complex (Figures 6c,d, Table 3).

## 4 Discussion

HD is a progressive, autosomal dominant neurodegenerative disorder characterised by motor dysfunction, cognitive decline, and psychiatric disturbances. The pathogenesis of HD involves oxidative stress, mitochondrial dysfunction, excitotoxicity, and neuroinflammation. Therapies for HD are still largely palliative, thus emphasizing the need for interventions that modify the course of the disease (Gatto et al., 2020; Paz-Rodríguez et al., 2024). The current research studies have centred their attention on natural substances exhibiting neuroprotective properties. The sesame seeds (*Sesamum indicum*) are widely grown in the tropics and subtropics, and they are characterized by the high content of the bioactive lignans – sesamin, sesamol, and sesamolin. These substances show significant antioxidant, anti-inflammatory, and neuroprotective activity (Javed et al., 2024; Ruankham et al., 2021). The relevance and novelty of the use of the extracts from the sesame seeds in neuroscience and the studies of neurodegenerative diseases can be attributed to the unique composition of bioactive components, neuroprotective effects, and unexplored possibilities in disorders such as HD, Alzheimer’s disease, and Parkinson’s disease. Lignans and particularly sesamol have shown considerable effectiveness in decreasing neurodegeneration. In the model of HD, sesamol reduced the neurotoxic effect of 3-nitropropionic acid through the increase of the activities of the mitochondrial enzymes and reduction of the oxidative stress markers and thus improved motor functions and body weight maintenance (Kumar et al., 2010). Furthermore, sesamol has been found to reduce the oxidative stress and inflammation pathways in the models of HD, thus suggesting its effectiveness as a drug. Moreover, it has been found that the sesame lignans such as sesamin and sesamol protect neurons from hydrogen peroxide-induced oxidative stress via regulating the SIRT1-SIRT3-FOXO3a pathway and inhibit cell death and promote survival (Sachdeva et al., 2015; Shyam et al., 2026; Singh et al., 2023).

After soaking 100 g of sesame seed powder in 300 mL of methanol for 7 days, the mixture was filtered and condensed at 40 degrees Celsius using a rotary evaporator. For compound identification, the crude extract was examined using LC-MS/MS (Q-Exactive Plus Orbitrap, IIT Bombay) in both positive and negative ESI modes. Phytochemicals with >95% confidence were screened through SwissADME for pharmacokinetic properties and ProTox 3.0 for toxicity. Eight drug-like, non-toxic compounds from sesame seed extract were selected based on SwissADME and ProTox 3.0 analyses. These were prepared using Open Babel and docked against four Huntington’s disease targets (HTT-17Q, Caspase-6, PDE10A2, SIRT1) using AutoDock Vina. Top complexes underwent 200 ns molecular dynamics simulations (GROMACS 24.1), followed by RMSD, RMSF, SASA, Rg, and H-bond analysis. Binding free energies were calculated using MM-PBSA (gmx_MMPBSA) from the final 10 ns of simulation to assess complex stability.

The results of this study demonstrated that the methanolic extract of sesame seeds, obtained through cold maceration, yielded 10.38 g/kg w/w and was subjected to LC-MS/MS analysis, identifying 91 and 74 compounds in negative and positive ionization modes, respectively, with 86 compounds showing high-intensity signals and >95% identification accuracy. ADMET predictions shortlisted 14 compounds with high GI absorption and BBB permeability, and eight of these compounds exhibited no toxicity across multiple filters. Molecular docking using AutoDock Vina revealed that while none of the eight compounds outperformed reference ligands on PT1 and PT2 targets, four compounds, particularly Valdiate (PubChem ID: 129715809), showed superior binding affinities on PT3 (−7.3 kcal/mol) and PT4 (−8.9 kcal/mol) compared to their respective references. Valdiate was further analysed through MDS using GROMACS for 200 ns, showing better protein and ligand RMSD, RMSF, SASA, and binding free energy on PT3, and comparable performance on PT4. Despite the fact that SwissParam generates CHARMM-consistent parameters for ligands, they still rely on the automation of the process and do not account for all the unique properties of the ligands. Nonetheless, the method of protein-ligand MDS has been used extensively for comparative studies and has enabled sufficient quality information on the stability of binding to be obtained. Notably, Valdiate exhibited greater stability than the reference compound on PT3 (P-RMSD: 0.269 nm vs. 0.347 nm; L-RMSD: 1.124 nm vs. 15.311 nm) and showed more favourable binding energy (−23.22 ± 2.45 kcal/mol vs. 0.00), while also achieving higher stability on PT4 as confirmed by its lower binding free energy (−28.87 ± 2.28 kcal/mol vs. −19.80 ± 3.68 kcal/mol).

“Valdiate” is categorised within the class of Valepotriates, which includes essential bioactive compounds such as valtrate that are integral to the pharmacological profile of *Valeriana officinalis* (Valerianaceae). Valepotriates, some of which include valtrate and didrovaltrate, mainly localize in the roots and rhizomes of *V. officinalis*, which form the most important medicinal parts of the plant (Patočka and Jakl, 2010). The subterranean structures serve as primary repositories for the main bioactive iridoid ester pools, which are responsible for the sedative and anxiolytic activities traditionally associated with valerian (Becker et al., 2014). In traditional phytopharmaceutics, plant extracts are usually obtained from the dried roots and rhizomes of the plant because of their abundant phytochemical content. Additionally, recent biotechnological research proved that *in vitro* plant tissue culture methods, such as callus or hairy root cultures, may be used for valepotriates’ biosynthesis.

Valepotriates have exhibited significant therapeutic efficacy in the treatment of various neurological conditions, particularly those characterised by anxiety, insomnia, epilepsy, and depression (Das et al., 2021). The main mechanism of action for valepotriates’ medicinal activity is related to the regulation of the GABAergic pathway, which is considered as the main inhibitory neurotransmitter signaling in the CNS (Khom et al., 2007; Sahin et al., 2024). Compounds, including valerenic acid and valepotriates (such as valtrate), enhance GABAergic neurotransmission by functioning as positive allosteric modulators of GABA A receptors, inhibiting the reuptake of GABA and facilitating its release. Consequently, this results in a net augmentation of inhibitory tone, which diminishes neural excitability and fosters relaxation, sedation, and stabilisation of mood. As per our knowledge, no study dealt with Valdiate compound alone or in combination for any neurodegenerative disease, but there were a few studies where the extract of plants, such as *V. officinalis,* was considered for neurological disease, as stated before. This marks the significance and the novelty of our study. However, Valdiate may be used in future studies for the *in vitro* and *in vivo* evaluations of HD and other neurological studies.

Interestingly, the detection of Valdiate in the methanolic extract of *S. indicum* represents a potentially novel phytochemical observation. Although sesame seeds are well known to contain lignans (e.g., sesamin, sesamolin, sesamol), phenolic acids, flavonoids, tocopherols, phytosterols, and unsaturated fatty acids. Valdiate has not been previously reported in *S. indicum* to best of or knowledge. The finding expands the known phytochemical profile of sesame and warrants further investigation. Future studies using an authentic reference standard and complementary structural characterization techniques such as NMR spectroscopy would further validate its identification.

## 5 Conclusion

This study proposed the therapeutical potential of *S. indicum* seed extract for HD treatment, emphasizing the bioactive constituent, Valdiate. From 86 identified compounds revealed by LC-MS/MS analysis, eight were selected through *in silico* ADMET screening, and eight compounds were determined as the best candidates for hit-to-lead process. Molecular docking demonstrated the highest binding affinity of the compound towards HD-related targets PDE10A2 (PT3) and SIRT1 (PT4). Moreover, the study used molecular dynamics simulation for 200 ns to prove the stability of protein Valdiate interactions. This research is innovative since it is the first one to report Valdiate’s ability to act as a multitarget ligand for treating HD. The compound shows favourable pharmacological and binding properties to be considered for further preclinical studies. All the findings will contribute to a better understanding of Valdiate’s role in HD treatment and help to perform further *in vitro* and *in vivo* experiments to explore its therapeutical potential as a multitarget HD treatment option.

## Data Availability

The raw data has been deposited to the GitHub repository: https://github.com/mukulshyam/Sesamum_indicum_valdiate.
