# Supplementary material for: Sesamum indicum-derived valdiate as a novel neuroprotective agent targeting PDE10A2 and SIRT1 in Huntington’s disease
Source: Front Bioinform. 2026 Aug 24;6:1868337. doi: 10.3389/fbinf.2026.1868337 (PMC13547276; doi:10.3389/fbinf.2026.1868337)
Supplement: Supplementary file 1 [file DataSheet1.pdf]

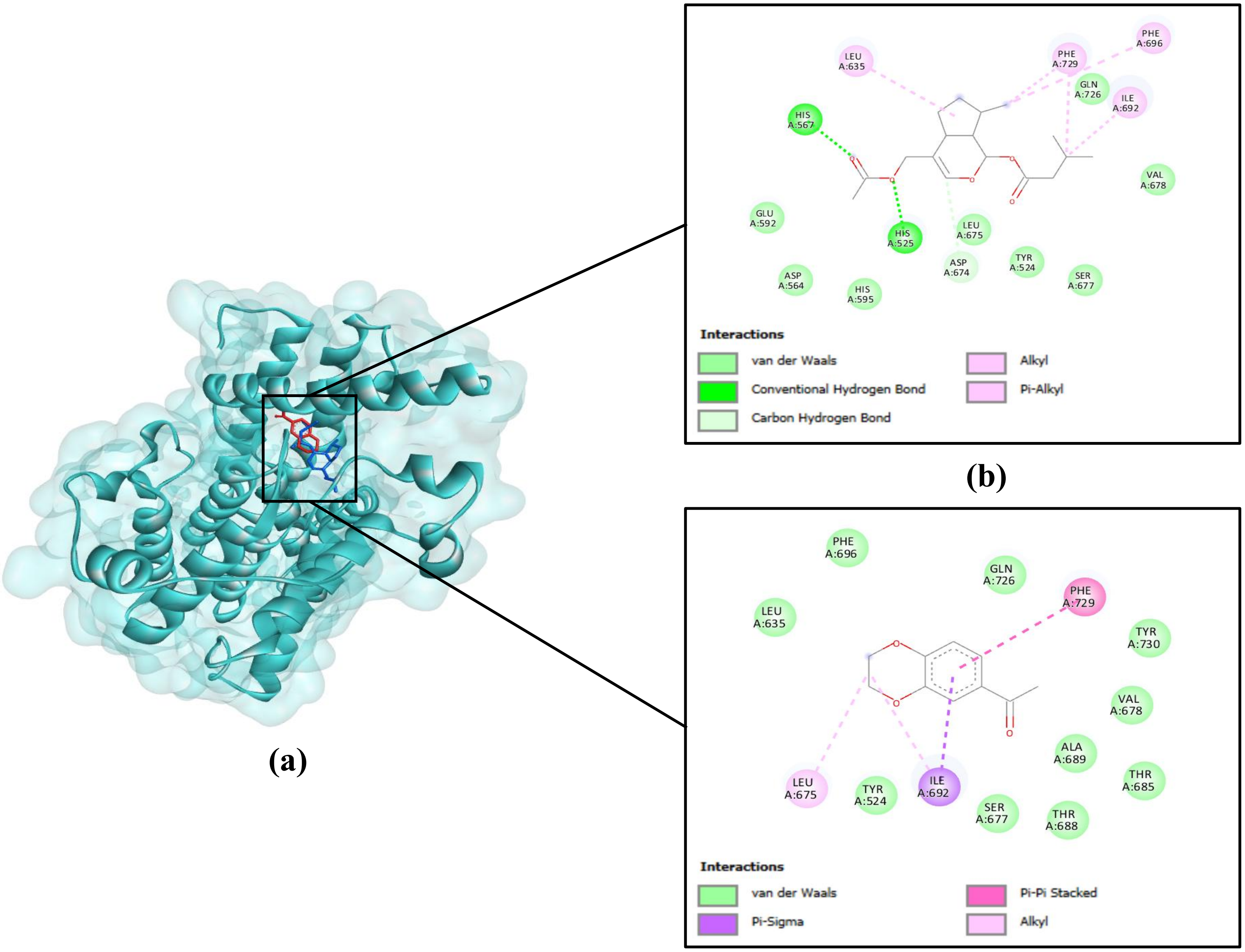
LEU A:635
PHE A:729
PHE A:696
GLN A:726
ILE A:692
HIS A:567
VAL A:678
GLU A:592
HIS A:525
LEU A:675
ASP A:674
TYR A:524
SER A:677
ASP A:564
HIS A:595
Interactions
van der Waals
Conventional Hydrogen Bond
Carbon Hydrogen Bond
Alkyl
Pi-Alkyl
PHE A:696
GLN A:726
PHE A:729
LEU A:635
TYR A:730
VAL A:678
ALA A:689
THR A:685
LEU A:675
TYR A:524
ILE A:692
SER A:677
THR A:688
Interactions
van der Waals
Pi-Sigma
Pi-Pi Stacked
Alkyl

(a)

(b)

(c)

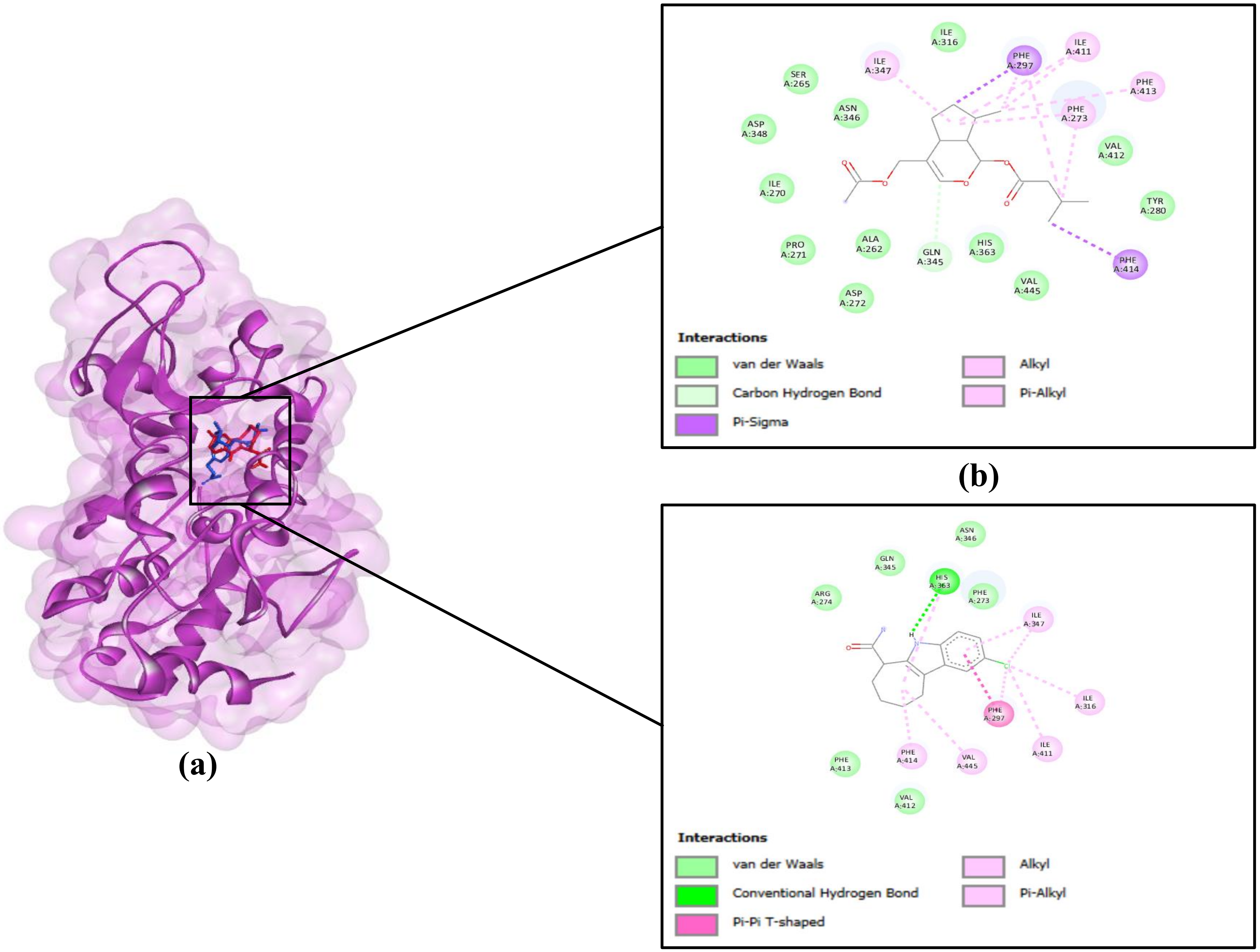
ILE A:316
PHE A:297
ILE A:411
ILE A:347
SER A:265
PHE A:413
ASN A:346
PHE A:273
ASP A:348
VAL A:412
ILE A:270
TYR A:280
PRO A:271
ALA A:262
GLN A:345
HIS A:363
PHE A:414
VAL A:445
ASP A:272
Interactions
van der Waals
Alkyl
Carbon Hydrogen Bond
Pi-Alkyl
Pi-Sigma
ASN A:346
GLN A:345
HIS A:363
ARG A:274
PHE A:273
ILE A:347
ILE A:316
PHE A:297
PHE A:414
VAL A:445
ILE A:411
PHE A:413
VAL A:412
Interactions
van der Waals
Alkyl
Conventional Hydrogen Bond
Pi-Alkyl
Pi-Pi T-shaped

(a) **(b)** **(c)**

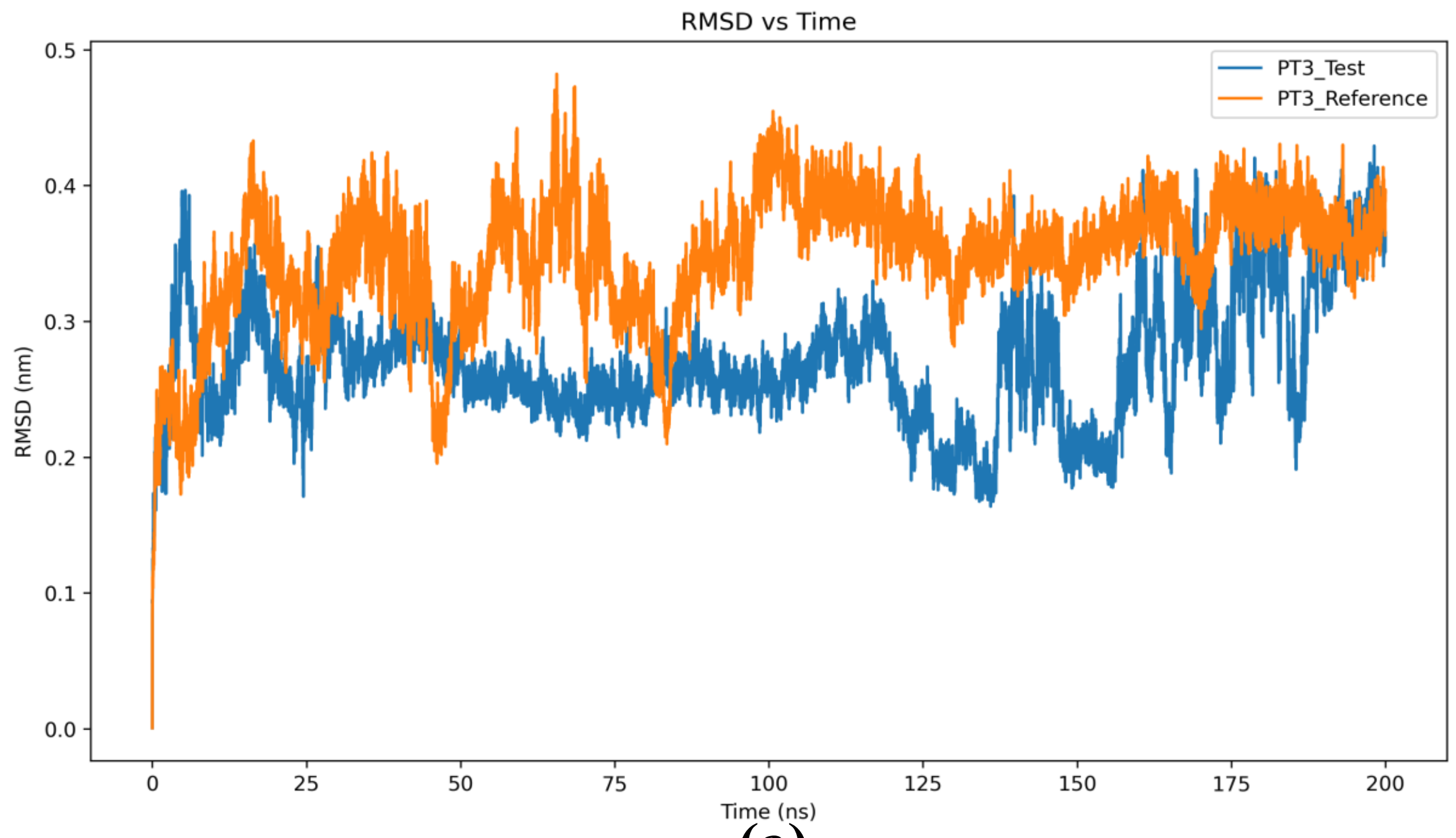
RMSD vs Time
RMSD (nm)
Time (ns)
PT3_Test
PT3_Reference
0.0
0.1
0.2
0.3
0.4
0.5
0
25
50
75
100
125
150
175
200

(a)

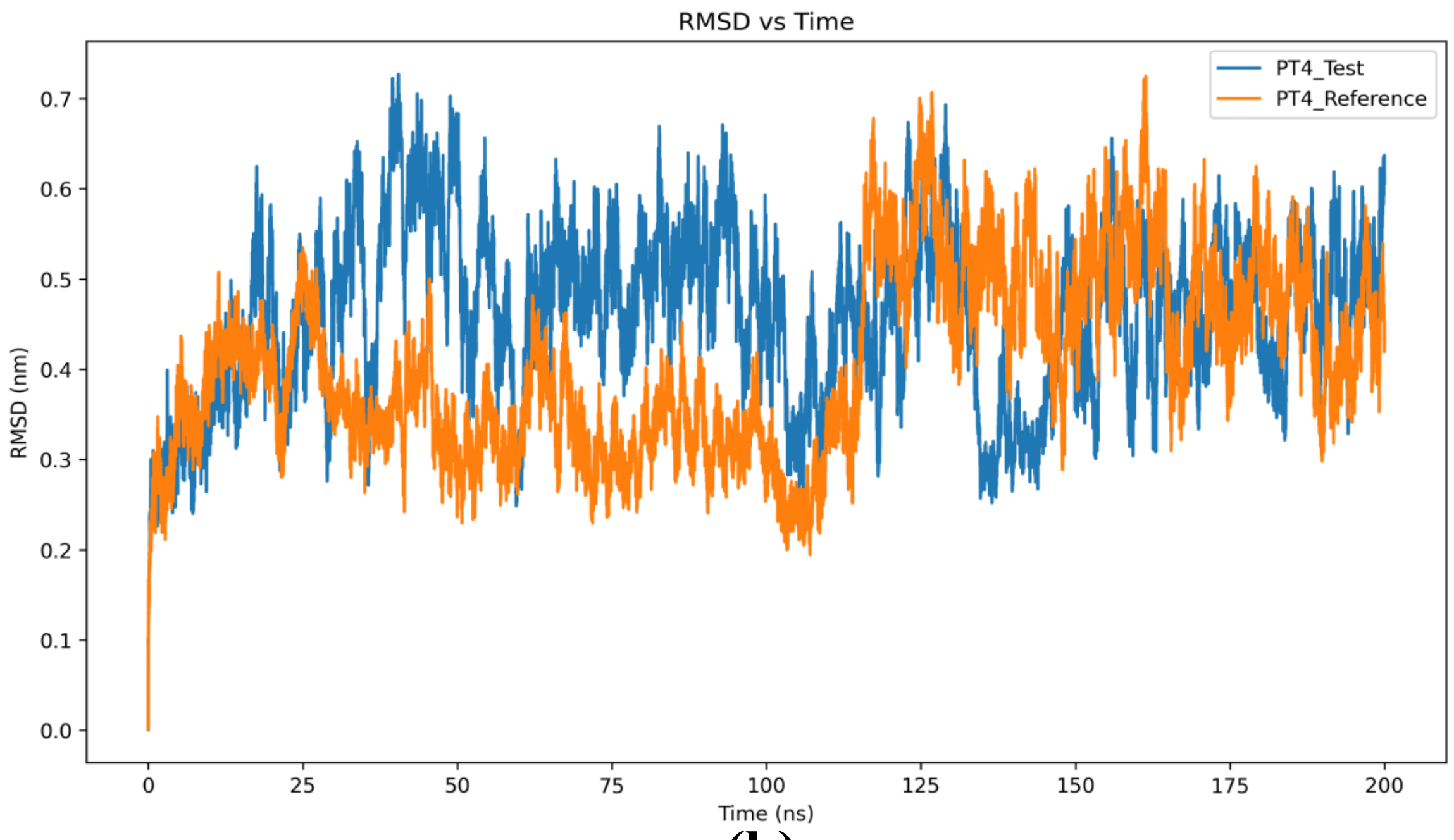
RMSD vs Time
RMSD (nm)
Time (ns)
PT4_Test
PT4_Reference
0.0
0.1
0.2
0.3
0.4
0.5
0.6
0.7
0
25
50
75
100
125
150
175
200

(b)

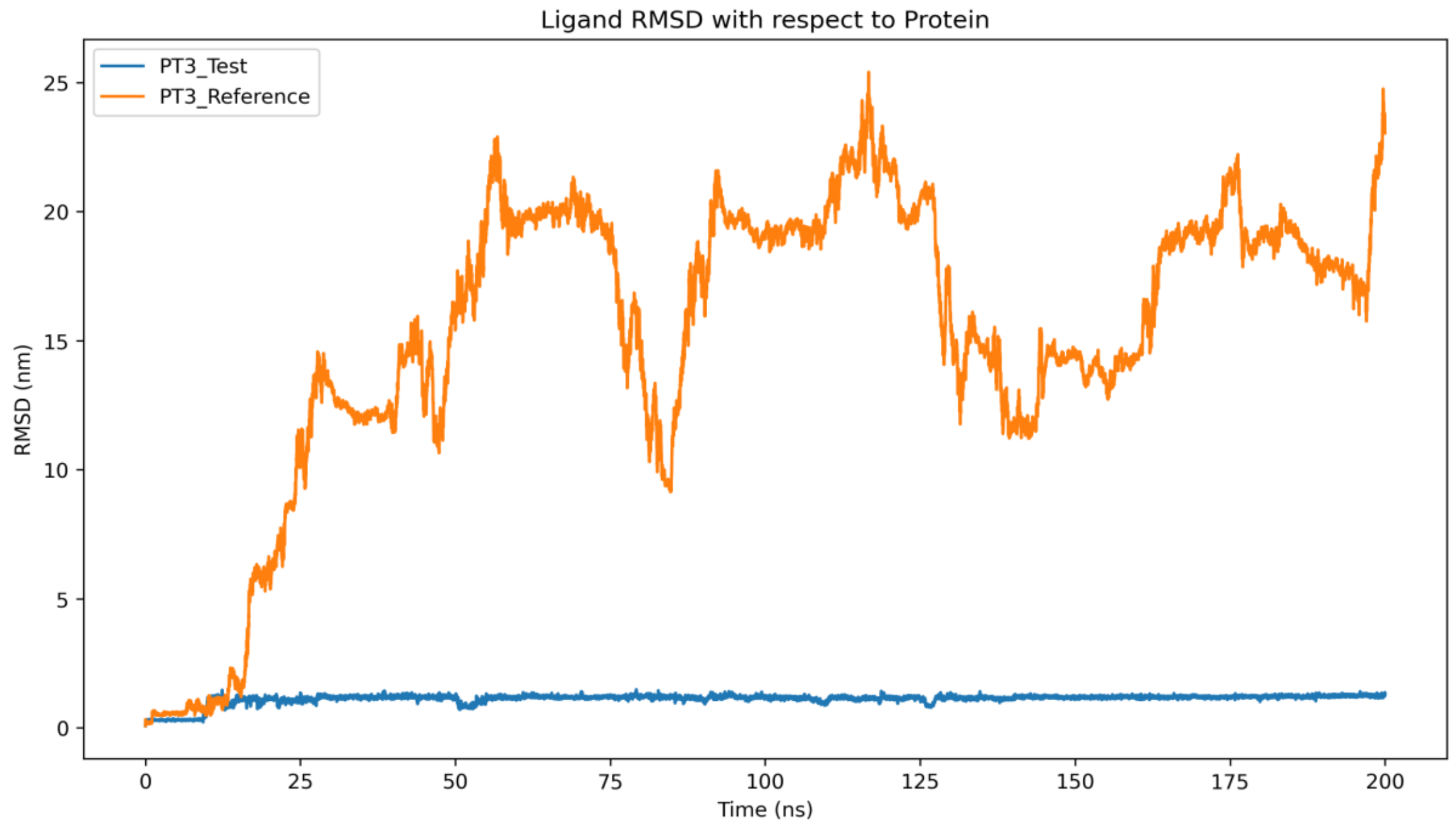
Ligand RMSD with respect to Protein
RMSD (nm)
Time (ns)
PT3_Test
PT3_Reference
0
5
10
15
20
25
0
25
50
75
100
125
150
175
200

(c)

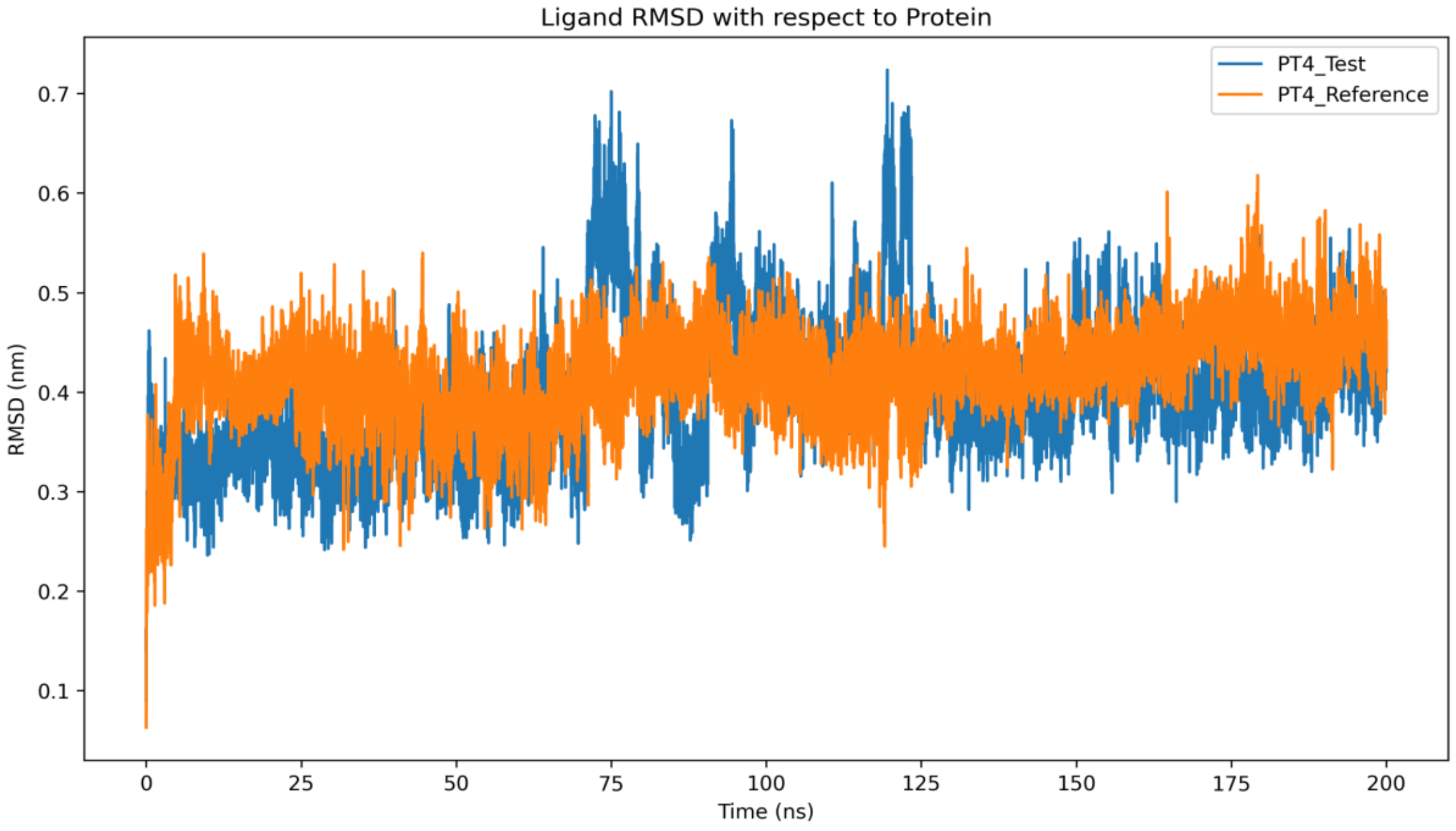
Ligand RMSD with respect to Protein
RMSD (nm)
Time (ns)
PT4_Test
PT4_Reference
0.1
0.2
0.3
0.4
0.5
0.6
0.7
0
25
50
75
100
125
150
175
200

(d)

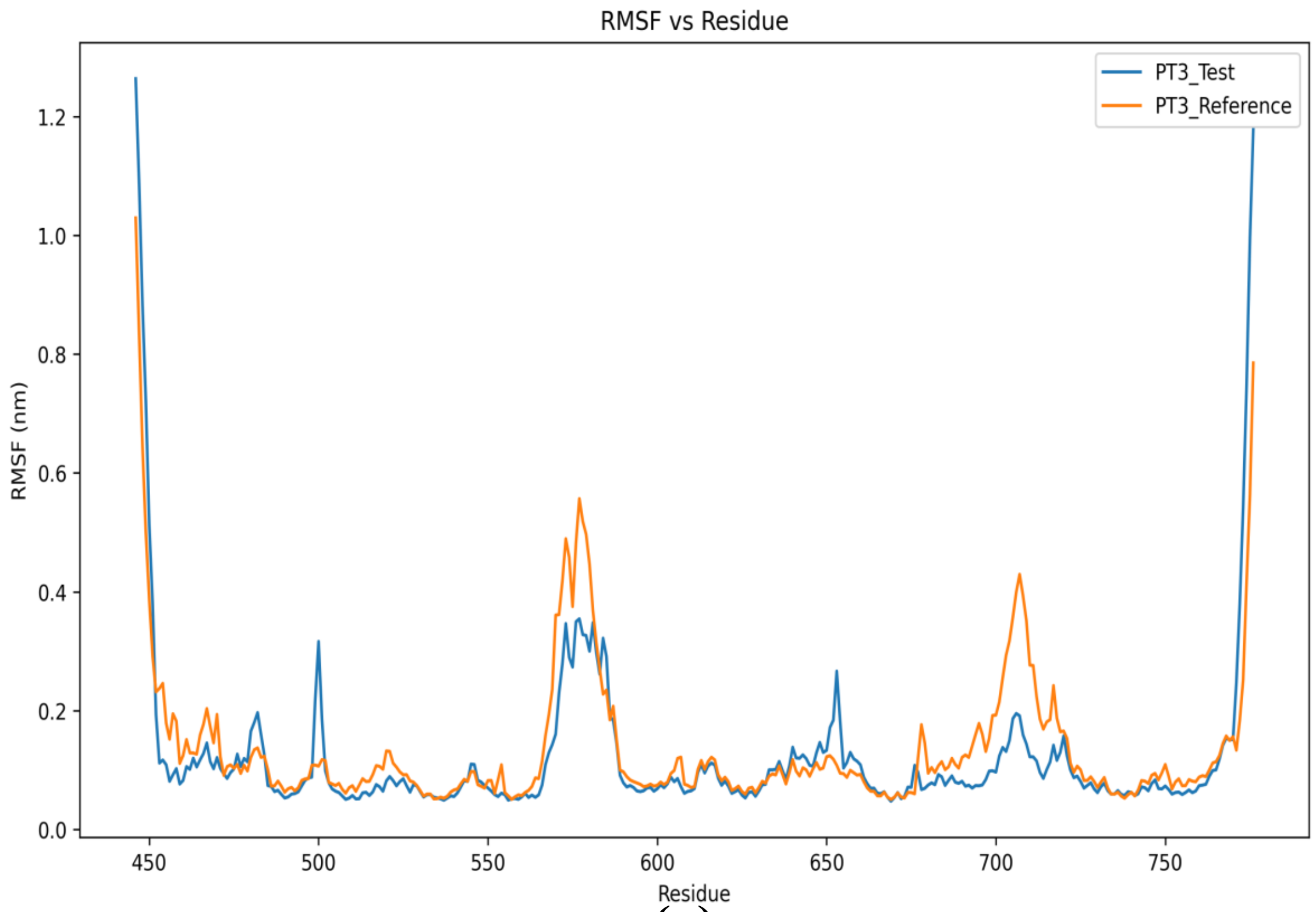

RMSF vs Residue
PT3_Test
PT3_Reference
RMSF (nm)
Residue
0.0
0.2
0.4
0.6
0.8
1.0
1.2
450
500
550
600
650
700
750

(a)

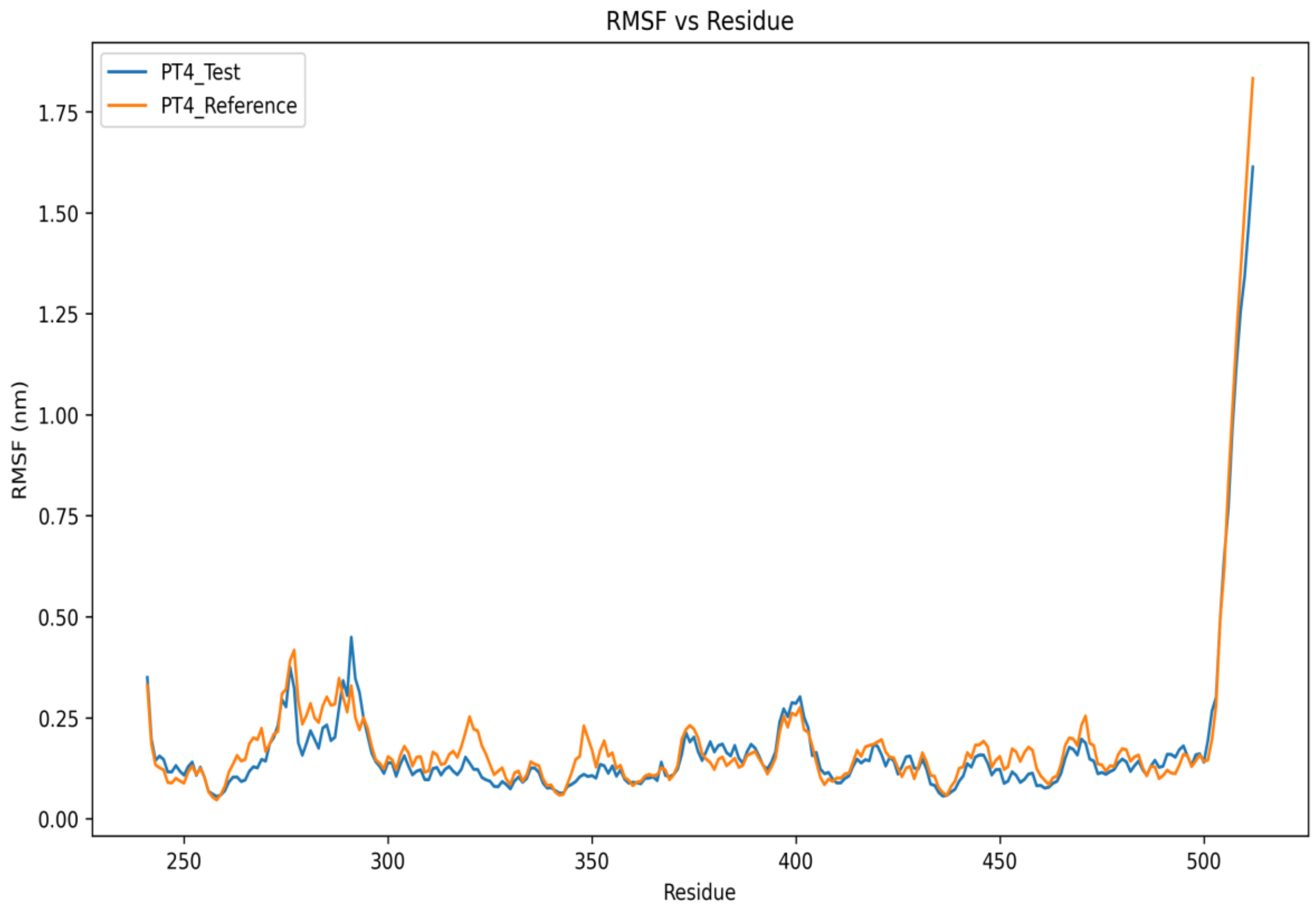

RMSF vs Residue
PT4_Test
PT4_Reference
RMSF (nm)
Residue
0.00
0.25
0.50
0.75
1.00
1.25
1.50
1.75
250
300
350
400
450
500

(b)

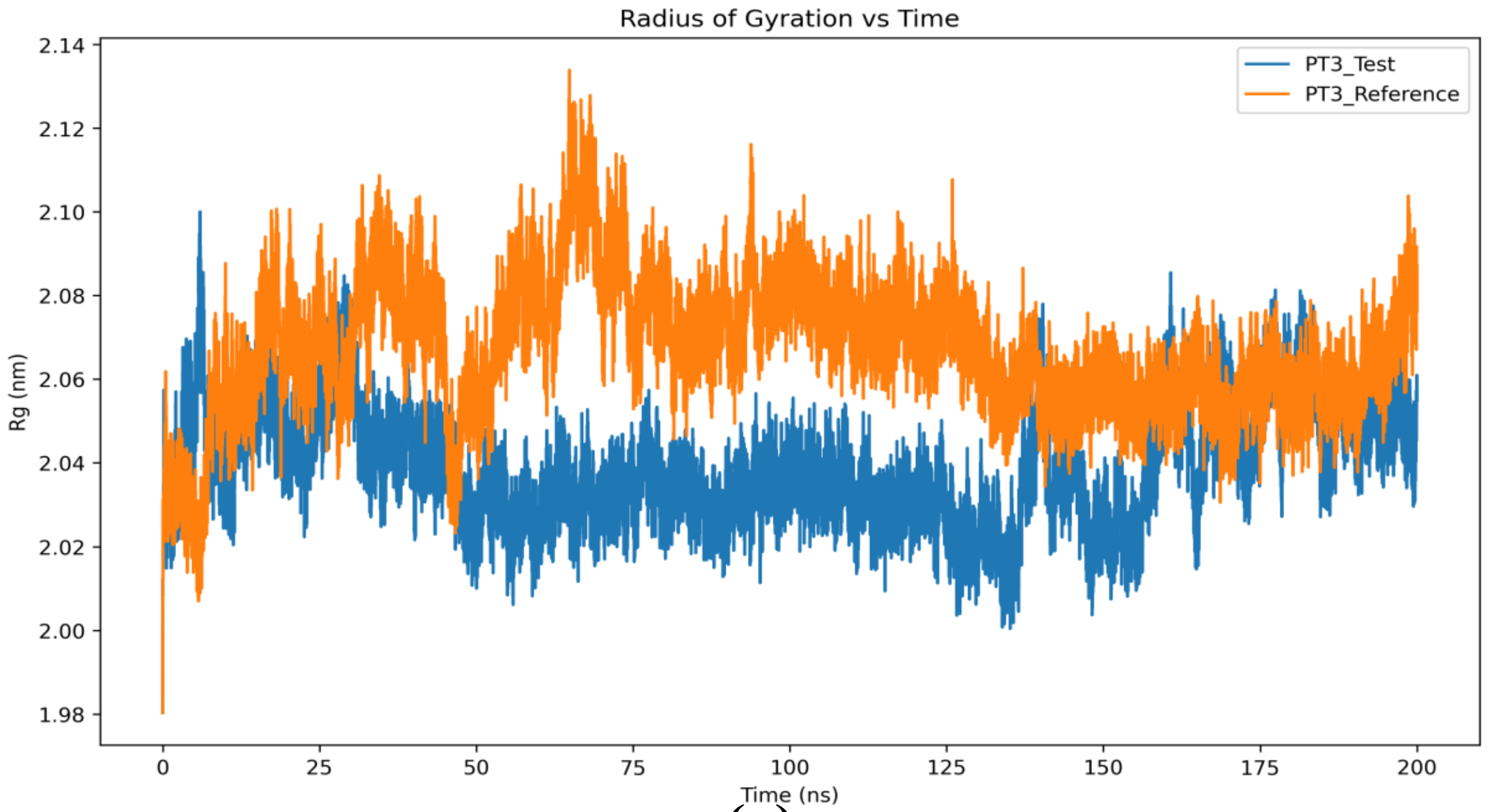
Radius of Gyration vs Time
Rg (nm)
Time (ns)
PT3_Test
PT3_Reference

(a)

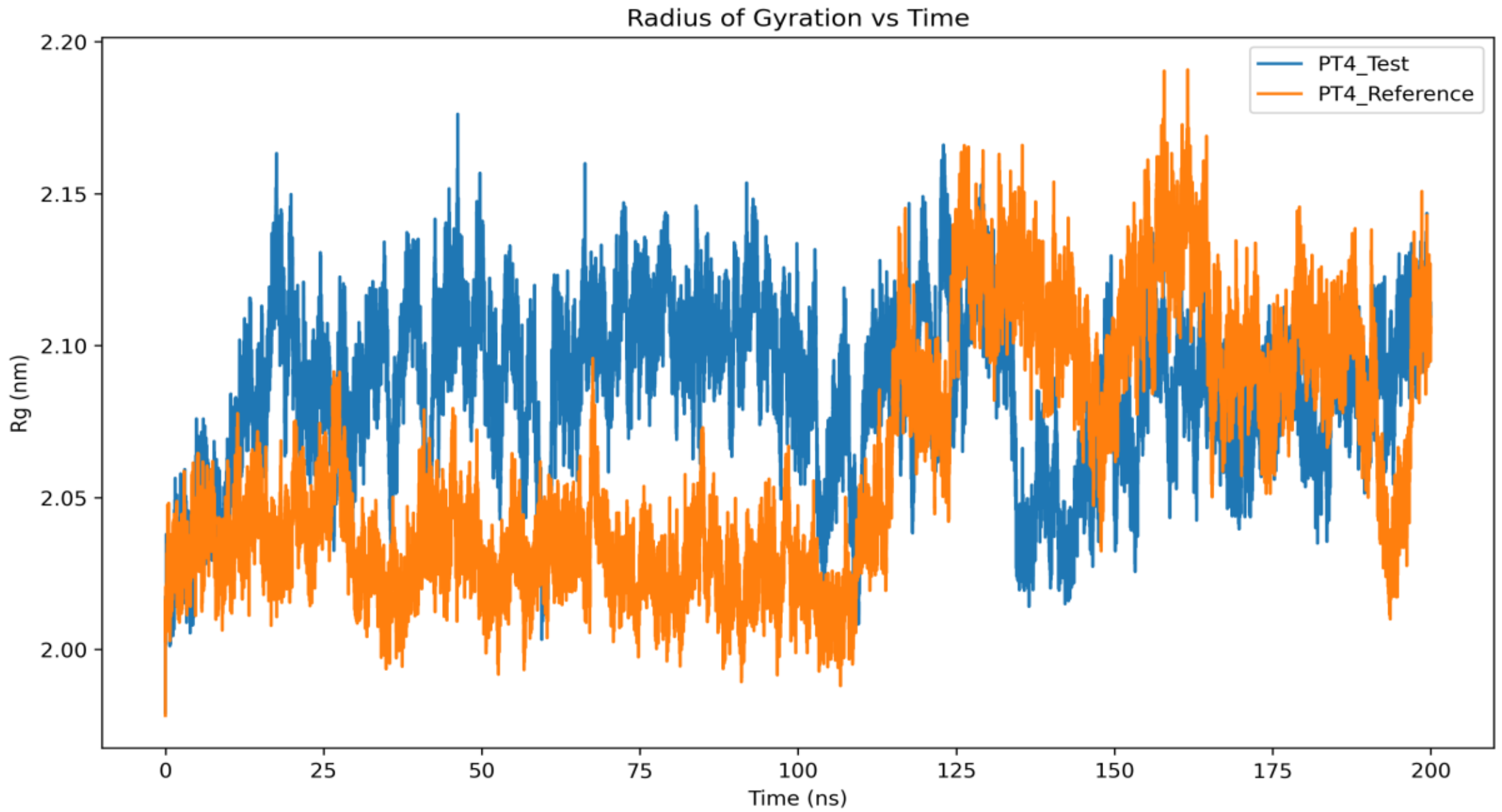
Radius of Gyration vs Time
Rg (nm)
Time (ns)
PT4_Test
PT4_Reference

(b)

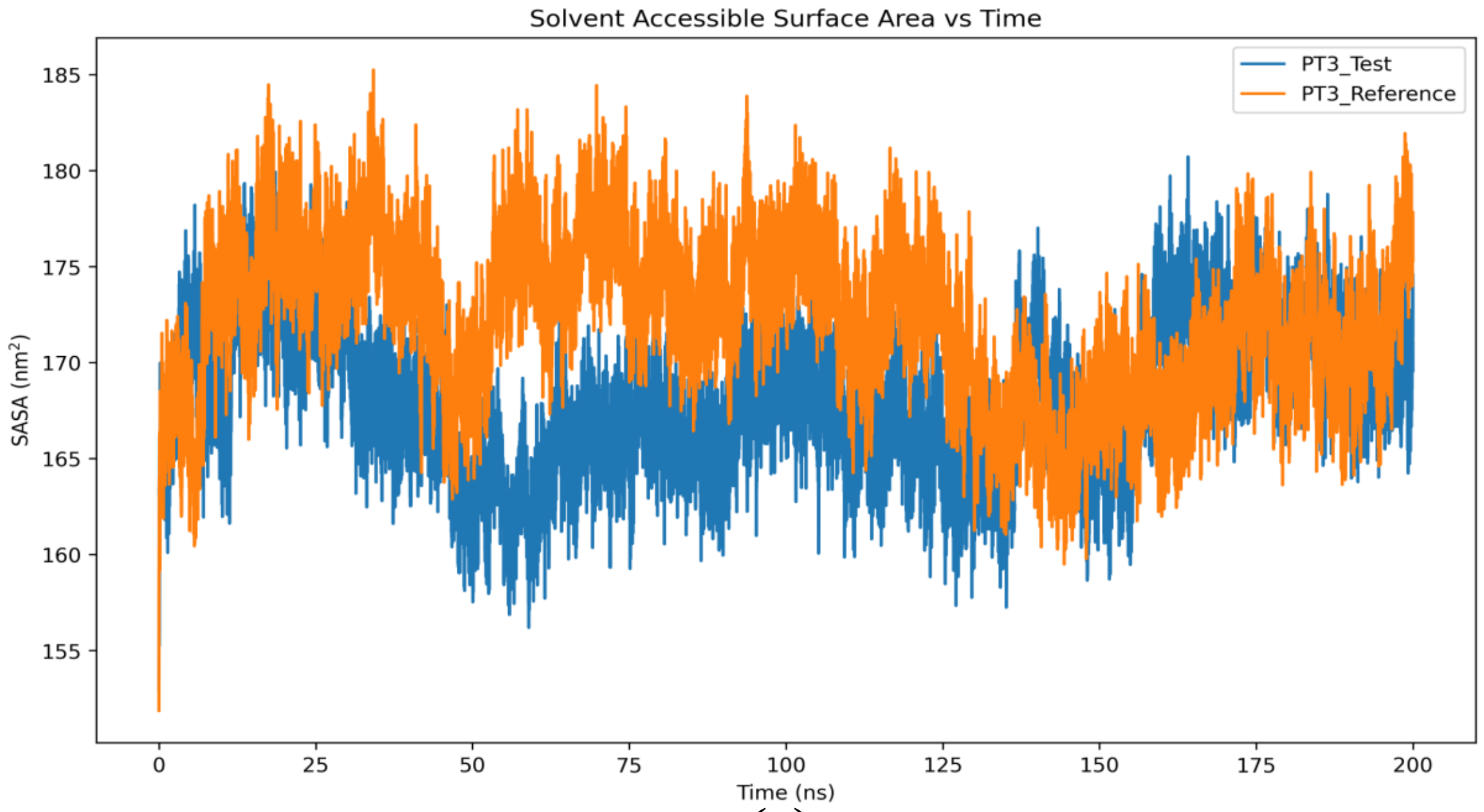
Solvent Accessible Surface Area vs Time
SASA (nm²)
Time (ns)
PT3_Test
PT3_Reference

(c)

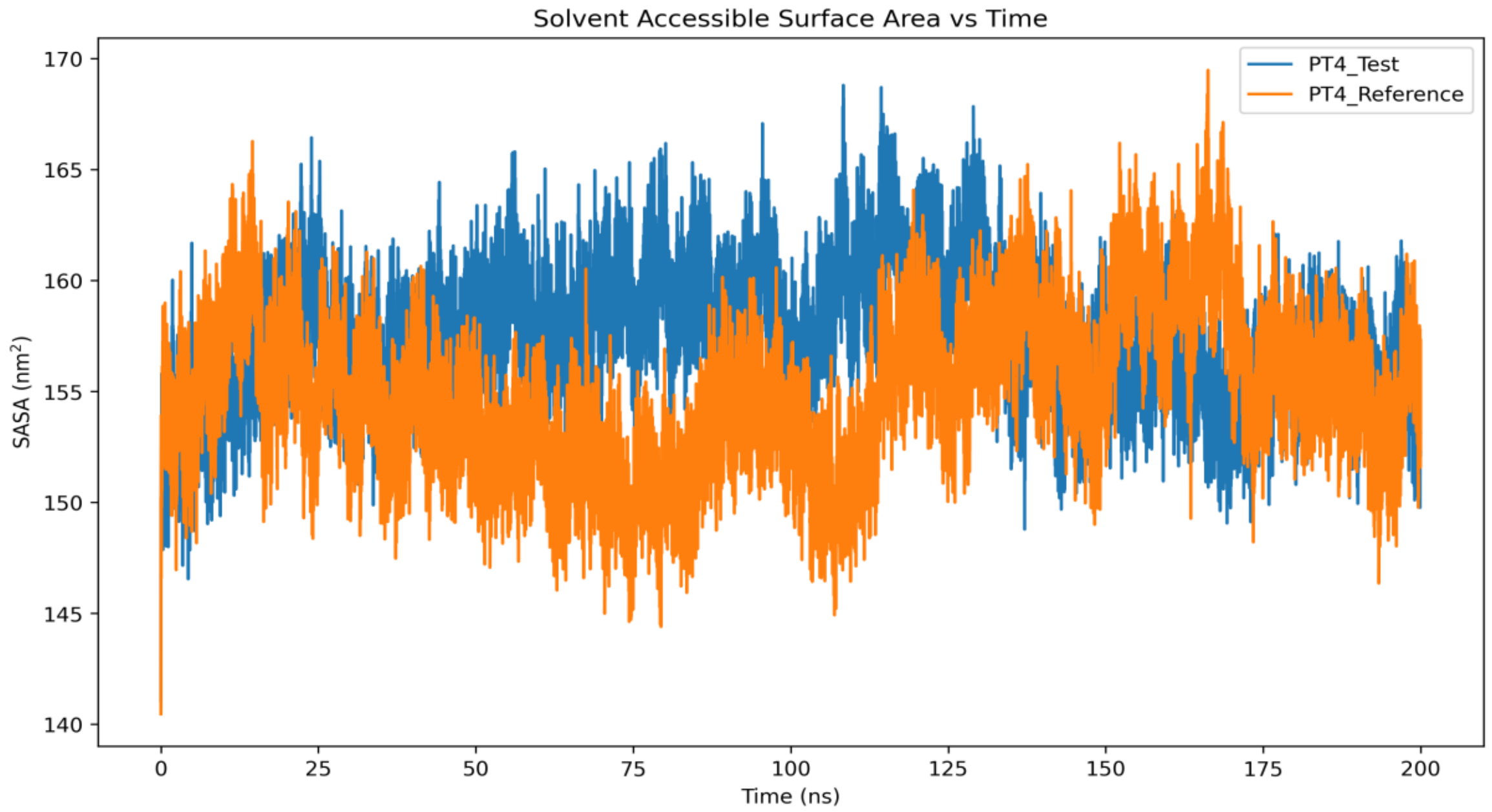
Solvent Accessible Surface Area vs Time
SASA (nm²)
Time (ns)
PT4_Test
PT4_Reference

(d)

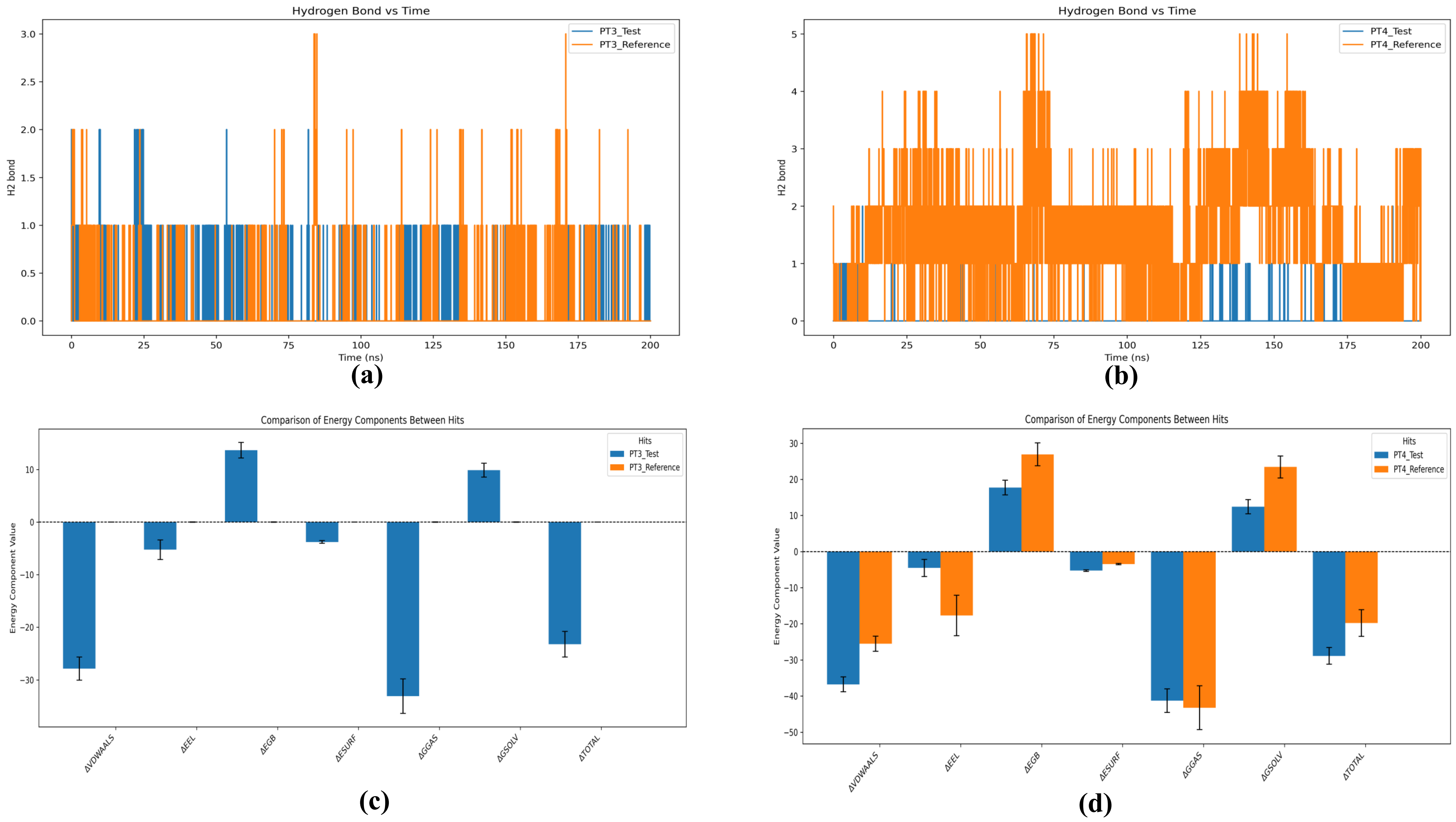

Hydrogen Bond vs Time
PT3_Test
PT3_Reference
H2 bond
Time (ns)
(a)
Hydrogen Bond vs Time
PT4_Test
PT4_Reference
H2 bond
Time (ns)
(b)
Comparison of Energy Components Between Hits
Hits
PT3_Test
PT3_Reference
Energy Component Value
ΔVDWAALS
ΔEEL
ΔEGB
ΔESURF
ΔGGAS
ΔGSOLV
ΔTOTAL
(c)
Comparison of Energy Components Between Hits
Hits
PT4_Test
PT4_Reference
Energy Component Value
ΔVDWAALS
ΔEEL
ΔEGB
ΔESURF
ΔGGAS
ΔGSOLV
ΔTOTAL
(d)
