# Supplementary material for: Sesamum indicum-derived valdiate as a novel neuroprotective agent targeting PDE10A2 and SIRT1 in Huntington’s disease
Source: Front Bioinform. 2026 Aug 24;6:1868337. doi: 10.3389/fbinf.2026.1868337 (PMC13547276; doi:10.3389/fbinf.2026.1868337)
Supplement: Supplementary file 2 [file Table1.docx]

***Sesamum indicum*-Derived Valdiate as a Novel Neuroprotective Agent Targeting PDE10A2 and SIRT1 in Huntington’s Disease**

Mukul Shyam, MD Wafi Ismail, Deepak Sharma, Prathap Srirangan, Rajiniraja Muniyan, Sabina Evan Prince^*^

School of Bio Sciences and Technology, VIT University, Vellore, India

**Table S1** Swiss ADME predictions

| PubChem ID | Compound Name | GI absorption | BBB permeant | Lipinski violations | Ghose #violations | Veber #violations | Egan #violations | Muegge #violations | PAINS #alerts |
| --- | --- | --- | --- | --- | --- | --- | --- | --- | --- |
| 442535 | Gentianamine | High | Yes | 0 | 0 | 0 | 0 | 0 | 0 |
| 11822857 | Prosolanapyrone II | High | Yes | 0 | 0 | 0 | 0 | 0 | 0 |
| 443437 | N-heptanoyl-homoserine lactone | High | Yes | 0 | 0 | 0 | 0 | 0 | 0 |
| 85890320 | 3,4',5,6,8-Pentamethoxyflavone | High | Yes | 0 | 0 | 0 | 0 | 0 | 0 |
| 77487 | Monomenthyl succinate | High | Yes | 0 | 0 | 0 | 0 | 0 | 0 |
| 15643 | Diamidafos | High | Yes | 0 | 0 | 0 | 0 | 0 | 0 |
| 66401 | 16alpha-Bromo-17beta-estradiol | High | Yes | 0 | 0 | 0 | 0 | 0 | 0 |
| 129715809 | Valdiate | High | Yes | 0 | 0 | 0 | 0 | 0 | 0 |

**Table S2** Toxicity evaluation using Protox web server

| PubChem ID | Compound Name | Hepatotoxicity | Neurotoxicity | Carcinogenicity | Cytotoxicity |
| --- | --- | --- | --- | --- | --- |
| 442535 | Gentianamine | Inactive | Inactive | Inactive | Inactive |
| 11822857 | Prosolanapyrone II | Inactive | Inactive | Inactive | Inactive |
| 443437 | N-heptanoyl-homoserine lactone | Inactive | Inactive | Inactive | Inactive |
| 85890320 | 3,4',5,6,8-Pentamethoxyflavone | Inactive | Inactive | Inactive | Inactive |
| 77487 | Monomenthyl succinate | Inactive | Inactive | Inactive | Inactive |
| 15643 | Diamidafos | Inactive | Inactive | Inactive | Inactive |
| 66401 | 16alpha-Bromo-17beta-estradiol | Inactive | Inactive | Inactive | Inactive |
| 129715809 | Valdiate | Inactive | Inactive | Inactive | Inactive |

**Table S3** Docking, type of bonds, and binding pocket

| **PubChem ID** | **Common name** | **PT1** | **Interactions** | **PT2** | **Interactions** | **PT3** | **Interactions** | **PT4** | **Interactions** |
| --- | --- | --- | --- | --- | --- | --- | --- | --- | --- |
| 11822857 | Prosolanapyrone II. | -7.1 | H Bond: HIS842, HIS802, HIS803, VD: ASP759, PHE812, PRO676, GLU677, GLY811, PRO800, PRO942, CYS813, ASP840, GLY974, GLU973, Other bonds: ASP934, PHE871, MET810, LEU943. | -5.9 | H bond: HIS219, C H Bond: CYS264, PHE263, VD: HIS121, SER218, HIS168, LYS265, GLU221, THR222, VAL261, Other bonds: TYR217, ARG220, CYS163 | -7.5 | H bond: SER677, C-H bond: TYR524, LEU675, VD: LEU635, ASP674, VAL678, THR685, THR688, ALA689, TYR693, GLN726, MET713, Other bonds: HIS525, HIS529, ILE692, PHE696, PHE729, | -6.2 | H Bond: CYS371, C H Bond: SER370, VD: ASN417, LEU418, GLU420, LYS408, LEU372, LYS375, GLY364, Other bonds: PRO419, VAL412, GLU416, GLU410. |
| 129715809 | Valdiate | -6.8 | H Bond: HIS802,  C H Bond: GLY811, VD: GLY974, ASP934, ASP840, HIS803, LEU943, PRO942, PRO676, ASP759, Other Bonds: PHE812, PHE871, HIS842 | -7 | H Bond: SER226, ARG220, VD: ARG65, THR67, GLN161, ASP70, GLY66, GLY225, HIS219, SER218, HIS168, GLU63, Other bonds: ARG64, TYR217, HIS121, PRO62 | -7.3 | H bond: HIS525, HIS567, C-H bond: ASP674, VD: TYR524, ASP564, GLU592, HIS595, LEU675, SER677, VAL678, GLN726, Other bonds: LEU635, ILE692, PHE696, PHE729 | -8.9 | C H Bond: GLN345, VD: SER265, ASN346, ILE316, VAL412, TYR280, VAL445, HIS363, ASP272, ALA262, PRO271, ILE270 ASP348, Other bonds: ILE347, ILE411, PHE297, PHE273, PHE413, PHE414. |
| 15643 | Diamidafos | -6.5 | H Bond: GLY811, HIS803, C H Bond: HIS842, VD: GLU677, PRO676, PHE871, PHE812, HIS802, PRO800, GLY974, GLY975, HIS976, Other Bonds: LEU943, ASP934, ASP840. | -5.6 | H Bond: ARG64, C H Bond: SER226, SER120, VD: PRO62, HIS121, CYS163, ALA162, SER218, HIS219, Other bonds: ARG220, GLN161. | -5.3 | H bond: ASP564, HIS567, GLU592, C-H bond: THR633, VD: TYR524, HIS563, SER571, ASN572, MET591, ASP634, ASP674, LEU675, ILE692, Other bonds: HIS525, ASP564, GLU592, HIS595, LEU635 | -5.1 | H Bond: SER370, C H Bond: LYS408, VD: PRO419, ILE411, LYS377, LEU372, Other bonds: VAL412, GLU410, GLU416, LYS375. |
| 442535 | Gentianamine | -6.6 | H Bond: HIS803, HIS802, VD: PHE812, GLU677, ARG681, GLY974, GLU973, VYS813, PHE871, GLY811, Other Bonds: PRO676, LEU943, HIS976, PRO800, HIS842. | -5.3 | H Bond: ASP262, C H Bond: VAL261, VD: GLU221, PHE263, THR222, ARG259, ARG260, Other bonds: TRP227 | -5.9 | C H Bond: TYR524, VD: HIS525, ASP674, HIS529, Other bonds: PHE729, PHE696, ILE692, LEU675. LEU635 | -6.3 | H bond: ASN417, C H Bond: LEU372, PRO409, GLU416, VD: PHE413, ILE411, LYS377, LYS375, SER370, Other bonds: VAL412, LYS408, GLU410 |
| 443437 | N-heptanoyl-homoserine lactone | -6.5 | H bond: HIS803, HIS842, GLY975, VD: ASP759, PHE812, GLU677, ARG681, GLY974, LEU943, ASP934, ASP840, HIS802, GLY811, Other bonds: PHE871, PRO800. | -6 | H Bond: ARG64, C H Bonds: SER226, VD: HIS168, SER218, CYS163, HIS121, GLN161, GLY225, THR67, ARG65, Other bonds: TYR217, ARG220, HIS219. | -6.4 | H Bond: GLN726, C H Bond: HIS525, LEU675, TYR693, TYR524, THR688, SER677, THR685, TYR730, PHE729, LEU635, Other bonds: ALA689, VAL678, PHE696, ILE692. | -6.5 | H Bond: ALA262, ARG274, C H Bond: GLY261, SER441, VD: ASN346, ILE411, ILE347, PHE273, VAL412, GL345, HIS363, VAL445, Other bonds: PHE297. |
| 66401 | 16alpha-Bromo-17beta-estradiol | -7.4 | VD: GLY811, PRO800, GLY974, LEU943, PRO942, Other bonds: HIS842, PHE812, PHE871. | -7.5 | H Bond: CYS264, VD: ALA269, ASP266, HIS168, SER218, HIS219, SER226, GLN161, THR67, Other bonds: TYR217, ARG220, ARG64, HIS121. | -9.3 | H bond: GLN726,  VD: SER571, ASN572, GLU592, ASP634, VAL678, MET713, TYR730, Other bonds: HIS525, THR633, LEU635, LEU675, ILE692, PHE696, PHE729 | -6.7 | H Bond: ASP292, VD: ASP298, ALA295, GLN294, LYS444, VAL445, ARG446, Other bonds: PHE414. |
| 77487 | Monomenthyl succinate | -5 | H Bond: HIS803, HIS842, ASP840, VD: LEU943, PHE871, ASP934, GLY974, GLY811, GLU973, PRO800, Other bonds: PHE812, HIS802. | -5.4 | H Bond: ARG64, GLN161, C H Bond: SER218, VD: HIS219, SER226, ASP70, THR67, GLY225, GLY66, Other bomds: ARG220, ARG65. | -5.1 | H Bond: ASN526, HIS529, TYR524, THR688, LYS528, VD: PRO523, ASP691, SER677, THE532, LYS680, VAL684 | -4.6 | H Bond: ALA262, ARG274, VD: GLY263, GLY440, ASP272, SER441, GLY261, PHE273, GLN345, LEU443, SER442. |
| 85890320 | 3,4',5,6,8-Pentamethoxyflavone | -6.5 | H Bond: HIS842, C H Bond: PRO942, VD: ASP759, GLY811, MET810, GLY974, GLU677, HIS803, LEU943, Other bonds: PHE812, PHE871, PRO800. | -7.8 | H Bond: ARG64, HIS121, ARG220, VD: GLY225, GLN161, SER218, HIS219, HIS168, Other bonds: CYS163, PRO62, TYR217. | -8 | H bond: HIS567, GLN726, C-H bond: GLU592, VD: TYR524, SER571, ASN572, THR633, LEU635, ASP674, LEU675, TYR693, MET714, GLY725, Other bonds: HIS525, HIS567, MET591, ILE692, PHE696, MET713, PHE729 | -7.1 | H Bond: LYS375, C H Bond: GLU416, LYS408, GLU410, VD: SER370, SER365, GLY364, THR368, VAL412, GLU420, LEU418, ASN417, ARG303, LEU372, ILE299, Other Bonds: PRO419 |
| Co-crystallized ligand | (Reference) | -10.3 | H Bond: HIS842, VD: ASP759, GLY811, PHE812, PRO800, ARG681, GLU677, HIS976, PRO676, GLY974, GLU973, LEU943, GLY975, HIS803, ASP840, ASP934, Other bonds: PHE871, HIS802. | -9.4 | H Bond: ARG220, SER218, SER226, ARG64, CYS264, VD: GLY66, THR67, GLN161, HIS219, LYS265, ASP266, TYR217, HIS121, Other bonds: ASP70, GLY225, ARG65, CYS163, PRO62. | -6.7 | VD: Leu635, Val768, Thr685, Thr688, Ala689, Phe696, Gln726 Tyr730, Other bonds: LEU675, ILE692, PHE729 | -8.3 | H bond: HIS363, VD: ARG274, GLN345, ASN346, PHE273, VAL412, Other bonds: ILE347, PHE297, ILE316, ILE411, VAL445, PHE414. |

Note: All the docking scores have been considered in kcal/mol. Abbreviations: Conventional Hydrogen Bond (H-bond): , Carbon-Hydrogen bond (C-H bond): Pi-donor H-bond, Van der Waals (VW): , Pi bonds (Pi-sigma, Pi-Pi T-shaped, Pi-Pi stacked, Pi-alkyl, Alkyl, Pi-cation, Pi anion, Pi-sulphur, Amide-Pi-stacked, Pi-Lone pair ), Halogen bond, Other bonds (Unfavorable Bump, Unfavorable donner-acceptor, Unfavorable donner-donner, Unfavorable acceptor-acceptor, attractive charges, Sulphur -X, Salt bridge)
